# Numerical Simulation and Experimental Validation of the Preparation of Multi-Lobe Multi-Hollow Profiled Fibers

**DOI:** 10.3390/polym18202567

**Published:** 2026-10-18

**Authors:** Shiqun He, Pei Feng, Qiuyi Zhang, Shengze Wang, Chongchang Yang

**Affiliations:** 1 College of Mechanical Engineering, Donghua University, Shanghai 201620, China; 1189709@mail.dhu.edu.cn (S.H.); 2231247@mail.dhu.edu.cn (Q.Z.); wasz@dhu.edu.cn (S.W.); ycc@dhu.edu.cn (C.Y.); 2 Engineering Research Center of Advanced Textile Machinery, Donghua University, Shanghai 201620, China

**Keywords:** profiled fiber, three-hollow eight-lobe, moisture wicking, anti-adhesion, melt-spinning

## Abstract

A novel three-hollow eight-lobe profiled fiber was designed and prepared via melt spinning to simultaneously enhance moisture wicking, anti-adhesion performance, and structural stiffness for sportswear applications. The proposed in-line three-hollow arrangement increased the average moment of inertia by 50.8% and reduced the average deformation by 80.0% compared with the triangular arrangement. The eight-lobe structure increased the degree of deviation from roundness (*D_R_*) by 22.2% relative to a four-lobe design, from 38.3% to 46.8%. Finite element analysis guided the micro-hole design (slit width 0.05 mm, three unit divisions, gap 0.06 mm). Under optimized quenching conditions (air speed 1.76 m/s, initial height 20 mm, temperature 45.2 °C), the as-spun fiber exhibited a *D_R_* of 60.1% and a hollowness ratio of 0.9%. After simple drawing, the *D_R_* increased to 63.77%, the hollowness ratio was 8.5%, the tenacity was 2.19 ± 0.02 cN/dtex (approximately 302 MPa), and the elongation at break was 96.4 ± 5.1% (*n* = 10). In preliminary fabric-level tests, the three-hollow eight-lobe fabric showed a one-way transport index of 169.1, which was 20.7% higher than that of the eight-lobe hollow control; the adhesion force was 24.1% lower and the adhesion distance was 31.3% shorter; and the clo value was slightly higher (0.71 vs. 0.68). However, because the number of fabric specimens was limited and the two fabrics differed in thickness, these fabric-level results should be considered preliminary, and no statistical significance is claimed. These results demonstrate that the three-hollow eight-lobe structure can be successfully fabricated and has potential for moisture-wicking sportswear.

## 1. Introduction

Fabric comfort is a multidimensional evaluation system that covers three core aspects: thermal–moisture comfort, tactile comfort, and pressure comfort [1]. Among these, thermal–moisture comfort exerts the most pronounced influence on overall wearing comfort [2]. Within the microclimate created by the “skin–clothing–environment” system, thermal–moisture comfort can be further divided into thermal performance and moisture performance [3]. When the temperature difference between the surrounding environment and the skin is not significant, moisture performance becomes the dominant factor affecting wearing comfort [4]. Because the thermal conductivity of water is about 23 times that of still air, moisture retention can significantly reduce the thermal resistance of fabrics. Once exercise stops, the continuous evaporation of residual moisture removes a substantial amount of heat, which may readily induce hypothermia. Enhancing moisture-wicking performance is therefore a key approach to balancing heat and moisture in the sports microclimate. Moreover, under dynamic sweating conditions, pressure comfort and tactile comfort often interact [5] and are strongly influenced by moisture transfer. Studies have shown that when a fabric with poor moisture-wicking performance adheres to the skin, the coefficient of kinetic friction between the fabric and the skin increases nonlinearly [6]. The surface tension generated when sweat wets both the fabric and the skin can produce a pronounced sensation of wet-state adhesion. This adhesion discomfort not only restricts the range of motion and increases normal and tangential resistance to body movement, but also readily causes skin abrasions. In summary, improving the moisture-wicking performance of fabrics remains a fundamental requirement for enhancing sportswear comfort during sweating.

The cross-sectional shape of fibers is a key factor determining the wicking performance of profiled fibers and their assemblies. With the advancement of materials science and spinning technology, physical modification of fiber structure has emerged as a forefront and mainstream trend. By altering the spinneret orifice shape or by incorporating functional materials for surface etching during spinning, fibers with irregular cross-sections are transformed from traditional circular shapes to non-circular shapes with axial grooves. Profiled fibers intended for sportswear have evolved from simple geometric shapes to complex multi-lobe structures. Representative products include triangular fibers, DuPont’s Coolmax functional fibers, Toyobo’s CoolDry fibers, the I-COOL series developed by Dongkook Trading Co., Ltd. (Seoul, Republic of Korea) [7], and CoolPlus fibers developed by Zhongxing Co., Ltd., Taiwan, China. Among these, multi-lobe structures (e.g., Y-shaped, W-shaped, cruciform, and eight-lobe) are classic designs for enhancing physical moisture transport, typically possessing three or more lobe-like branches [8]. Their core design concept is to enlarge the specific surface area by increasing the number of lobes, thereby generating a multitude of non-circular, wedge-shaped capillary interstices with reduced curvature radii within the yarn. However, increasing the number of lobes alone often compromises structural stiffness and may lead to fiber collapse under compression [9].

Simultaneously, hollow fibers oriented toward light weight, thermal insulation, and stiffness constitute another important research direction. The design of hollow fibers draws inspiration from biomimicry of natural insulating fibers such as kapok and ramie [10]. With the development of alkali-soluble and water-soluble composite spinning technologies, multi-hollow fibers have advanced rapidly. From a mechanical perspective, compared to multi-hollow structures, traditional single-hollow structures are more prone to cross-sectional flattening deformation under compression, leading to diminished hollowness or even functional failure. Multi-hollow structures can improve the compression performance of fibers, allowing them to maintain stable channel morphology under wet or compressed conditions, providing guidance for the subsequent design of fiber cross-sections that combine high moisture conductivity and high stiffness. Recent studies have investigated multi-hollow fibers with triangular, square, and circular hollow arrangements for improved compressive resistance and thermal insulation [11,12]. Nevertheless, the combined effect of multiple lobes and multiple hollows on moisture wicking and anti-adhesion has rarely been systematically studied.

Despite these advances, existing multi-lobe hollow fibers face inherent conflicts. First, increasing lobe count improves wicking but reduces wall thickness and bending stiffness. Second, more hollow channels disrupt material continuity and create stress concentrations that weaken mechanical strength. Third, bilayer designs intended to separate wicking from load-bearing functions introduce interfacial defects and raise manufacturing costs. Consequently, few studies have systematically reconciled deep grooves for wicking, adequate stiffness for anti-adhesion, and collapse-resistant channels for sustained moisture transport.

To address this gap, a three-hollow eight-lobe fiber with an in-line hollow arrangement was developed. This design offers three key contributions: (1) combining eight lobes to maximize the capillary effect with three square hollows to balance axial and radial stiffness; (2) introducing an in-line hollow arrangement that, according to finite element analysis, increased the average moment of inertia by 50.8% and reduced the average deformation by 80.0% compared with the triangular configuration; and (3) establishing a forward-design framework that integrates mechanism analysis, numerical simulation, and experimental evaluation.

This study adopted a multi-scale approach to evaluate the proposed fiber. At the single-fiber scale, extrusion deformation, die swell, and expansion adhesion were simulated to verify spinnability. At the fiber-bundle scale, wicking and thermal insulation were predicted using a bundle unit model coupled with a random cross-sectional simulation system. At the fabric scale, preliminary tests were conducted in accordance with national standards. The overall research workflow is as follows. First, the moisture transport and anti-adhesion mechanisms were analyzed to identify the key cross-sectional parameters. Second, simplified three-dimensional models with four to eight lobes and one to seven hollow channels were established to determine the number of lobes, the number of hollow channels, and the hollow arrangement. Third, the spinneret micro-hole was designed, and its mechanical strength was evaluated by finite element analysis. Fourth, the melt flow, extrusion swell, and bonding behavior were simulated to verify spinnability. In addition, fiber-bundle-scale simulations were performed to predict the wicking and thermal performance of the candidate cross-sections. Fifth, melt-spinning experiments were conducted using the optimized spinneret, and the effects of quenching start height and cooling air velocity on cross-sectional formation were investigated. Sixth, the mechanical properties of the prepared fiber were characterized. Finally, the three-hollow eight-lobe fiber and the eight-lobe hollow control fiber were knitted into fabrics under identical conditions, and fabric-level moisture wicking, adhesion, and thermal insulation were evaluated. Fabric-level results were considered preliminary because of the limited number of independent specimens. This multi-scale approach explores the moisture-management potential of the proposed fiber and provides a theoretical and technical foundation for designing fibers with balanced stiffness, wicking, and anti-adhesion properties. Detailed methods and results are presented in the corresponding sections.

## 2. Mechanisms of Moisture Transport and Anti-Adhesion

### 2.1. Transport Mechanism

The ability of fabric to manage liquid sweat is the core factor determining the comfort of sportswear. From macroscopic fabrics to microscopic fibers, the conduction of water follows well-defined physical principles.

(1) Differential capillary effect at the macro level. Directional moisture transport refers to the phenomenon in which structural or wettability gradients across the fabric thickness drive liquid water from the inner layer (skin side) to the outer layer (air side) while preventing reverse flow. This mechanism effectively reduces the discomfort caused by prolonged sweat contact with the skin. The capillary driving force is described by the Laplace equation [13]:

(1) $$p=\frac{2\gamma \cos\theta }{R}$$

where γ is the liquid surface tension, θ is the contact angle, and R is the equivalent capillary radius. The capillary pressure p is a suction pressure; its absolute value increases with stronger capillary driving force.

When the fabric has different knitting densities on its two sides, the equivalent capillary radius on the skin side ($R_{2}$) is relatively large, whereas that on the air side ($R_{1}$) is small. This structural gradient generates an additional pressure difference Δp given by [14]:

(2) $$\Delta p=2\gamma \left(\frac{\cos\;\theta_{2}}{R_{2}}-\frac{\cos\;\theta_{1}}{R_{1}}\right)$$

where $\theta_{1}$ and $\theta_{2}$ are the contact angles of the air side and skin side, respectively. By designing the fabric such that Δp > 0, water is directionally transported from the inner to the outer side. The differential capillary effect described above has been experimentally validated at the fabric level. Ge et al. [15] demonstrated that a structural gradient in capillary radius across the fabric thickness enables unidirectional sweat transport, confirming that tailoring the equivalent capillary radius from the skin side to the air side is an effective strategy for directional moisture transport.

To quantitatively describe the above process, the fabric is abstracted as a one-dimensional, two-layer porous medium along the thickness direction, and a saturated–unsaturated flow model is established based on the structural gradient.

The governing equations consist of mass conservation and Darcy’s law, neglecting gravity and evaporation [16]:

(3) $$\frac{\partial \left(\varphi s\right)}{\partial t}+\frac{\partial u}{\partial x}=0$$

(4) $$u=-\frac{K}{\mu }\frac{\partial p}{\partial x}$$

where φ is the porosity, s is the saturation, u is the Darcy velocity, *K* is the permeability (related to *R*), μ is the dynamic viscosity of water, and p is determined by Equation (1). The model is based on the following assumptions: the fabric is a rigid skeleton with unchanged pore structure; the contact angle and equivalent radius are uniform within each layer, denoted as $\theta_{1}$, $R_{1}$ and $\theta_{2}$, $R_{2}$; the liquid water properties are constant; local capillary equilibrium holds instantaneously, neglecting dynamic contact angle effects; the fabric is sufficiently thin that gravitational gradients are neglected; and only liquid water transport is considered.

The computational representation of moisture transport proceeds as follows:

1. $p_{1}=2\gamma cos\theta_{1}/R_{1}{,p}_{2}=2\gamma cos\theta_{2}/R_{2}$. Since $R_{1}<R_{2}$, $\left|p_{1}\right|>\left|p_{2}\right|$, the air side possesses a stronger suction capacity.
2. The driving pressure difference across the interface between the two layers is precisely the additional pressure difference $\Delta p=p_{2}-p_{1}$, as expressed in Equation (2). When Δp>0, forward transport occurs; if flow attempts to reverse, the driving pressure difference becomes negative, naturally opposing the backflow.
3. The Darcy flux between nodes is discretized as $q=-\left(K_{eff}/\mu \right)(\Delta p_{node}/\Delta x)$, and the interfacial flux as $q_{int}=\left(K_{int}/\mu \right)(\Delta p/\delta )$, where $K_{eff}$ and $K_{int}$ can be taken as harmonic mean permeabilities and δ is the characteristic interfacial thickness.
4. The fluxes at each node are substituted into the discretized form of Equation (3), and the saturation field is updated explicitly or implicitly.

By repeating the above steps, the unidirectional transport of moisture from the skin side to the air side can be simulated, inherently reflecting the anti-reverse-flow property determined by the structural gradient. This model can serve as the foundation for a holistic simulation of fabric heat and moisture transfer.

(2) Fiber wicking effect at the microscopic level. Moisture transport in fabrics is a multiscale process involving both macroscopic pores between fiber bundles and microscopic capillaries formed among densely packed fibers within a fiber bundle. For a given fiber cross-section, the effective capillary radius between tightly packed fibers is not the macroscopic pore size, but is significantly influenced by the surface grooves. Through structural design, multi-lobe profiled fibers form multiple continuous and regular longitudinal grooves on the axial surface of the fibers, creating numerous low-resistance capillary channels at the micrometer level. Driven by surface tension, sweat rapidly and spontaneously transports along these directional grooves, achieving efficient unidirectional transfer and avoiding the cold, wet and sticky sensation caused by widespread skin wetting.

To quantify the influence of cross-sectional shape on capillarity, the degree of deviation from roundness *D_R_* is defined as shown in Equation (5):

(5) $$D_{R}=\frac{R-r}{R}\times 100\%$$

where R is the circumscribed circle radius of the fiber cross-section and *r* is the inscribed circle radius. A larger *D_R_* implies a more pronounced deviation from circularity and more prominent surface grooves, which generate stronger capillary forces with liquids and thus facilitate directional moisture transport. This can be accounted for by relating the equivalent capillary radius to the profile degree.

### 2.2. Anti-Adhesion Mechanism of Fabrics

(1) Liquid Bridge Force and Capillary Adhesion Theory

When the human body perspires, sweat displaces the air within the pores of the fabric. As the skin approaches the wetted fabric surface, sweat spontaneously forms numerous tiny liquid bridges between the skin and the fibers, creating liquid channels that connect the two phases [17]. Neglecting gravity, the shape of a liquid bridge can be described by the Young–Laplace equation:

(6) $$\Delta p=\gamma \left(\frac{1}{\rho_{1}}+\frac{1}{\rho_{2}}\right)$$

where Δp is the pressure difference across the liquid–vapor interface, *γ* is the surface tension of sweat, and $\rho_{1}$ and $\rho_{2}$ are the outer radius of curvature and the minimum inner radius of curvature of the bridge’s revolved surface, respectively.

The static liquid bridge force F arises from the combined contributions of the surface tension and the Laplace pressure difference, and is given by

(7) $$F=2\pi \gamma \rho_{2}+\pi \rho_{2}^{2}\Delta p$$

Because the separation between the fabric and the skin is small and the liquid bridge volume is limited, Equation (7) can be simplified to yield an approximate expression for the liquid bridge force [18]:

(8) $$F=2\pi \gamma Rcos\theta \left[1-\frac{S}{2\rho_{1}cos\theta }\right]$$

Here, R is the equivalent radius of the contact region between sweat and fiber, θ is the contact angle, and S is the local separation distance between the fabric and the skin.

Equation (8) provides the quantitative relationships that govern the adhesion force: the liquid bridge force *F* is approximately proportional to the equivalent contact radius R and inversely related to the separation distance S. This demonstrates that reducing the contact area and enlarging the fabric–skin separation can fundamentally weaken or even disrupt the liquid bridges.

The adhesion force between wetted fabric and skin tends to pull the fabric against the body surface during movement. Recent theoretical work has established a quantitative model for fabric wet adhesion based on the mechanical equilibrium of liquid bridges on fabric structures, demonstrating that wet adhesion can be regulated through structural design [19].

(2) Influence of Fabric Stiffness on Anti-Adhesion

Based on the liquid bridge theory outlined above, the fabric–skin adhesion force is governed by the equivalent contact radius R and the separation distance S. Fiber stiffness is the key mechanical factor that modulates these two geometric parameters: enhancing the bending stiffness and compressive stiffness of fibers can quantitatively destroy the conditions necessary for liquid bridge formation at the microscopic level.

(a) Altering the contact mode to reduce the equivalent contact radius *R*

Under the combined effects of sweat wetting and compressive forces during body movement, low-stiffness fibers are prone to buckling instability. This causes the fabric structure to collapse onto the skin, forming large-area conformal contacts with an equivalent contact radius R that can reach several times the fiber diameter or even approach the yarn scale. In such cases, the liquid bridges tend to become continuous, and the large R directly leads to high adhesion forces according to Equation (8). In contrast, high-stiffness fibers exhibit superior shape retention, converting the contact mode from conformal contact into discrete point or line contacts. The effective contact radius R is thus divided into numerous small values corresponding to the dimensions of fiber tips or micro-groove ridges.

(b) Maintaining a stable spatial structure to increase the separation distance *S*

The bending stiffness *EI* of a fiber bundle (*E* is the material modulus, *I* is the area moment of inertia of the cross-section) directly determines its deflection δ under wet and compressed conditions. For a simplified model under uniform pressure q, the deflection scales as $\delta \propto qL^{4}/(EI)$. Increasing I significantly reduces the deflection, preserving a stable micron-scale air gap $S=S_{0}-\delta$ ($S_{0}$ is the initial spacing) between the fabric and the skin, thereby suppressing the liquid bridge force. For fibers with the same cross-sectional area, adopting a multi-hollow structure can dramatically increase the area moment of inertia.

The multi-lobe, multi-hollow cross-section combines external grooves for enhanced capillary wicking with internal hollow channels to improve the structural performance of the fiber. This design reduces the liquid-bridge contact radius R by approximately an order of magnitude and maintains a larger fabric–skin separation S, thereby lowering the adhesion force. In contrast to conventional solid round fibers, which exhibit an inherent trade-off between wicking capability and stiffness, the proposed configuration enables concurrent improvement of both properties, effectively mitigating the clammy and sticky sensation associated with sweat-induced fabric adhesion.

### 2.3. Selection Rationale for Key Cross-Sectional Parameters

The fabrication of multi-lobe multi-hollow spinnerets is constrained by several geometric limits. Slit widths below 0.05 mm cause clogging and excessive pressure drops, while widths above 0.08 mm reduce lobe tip definition and weaken the capillary effect. The gap between adjacent slits must balance spinneret strength with melt bonding for hollow structure formation.

(1) Selection of the number of fiber lobes

**Figure 1:**
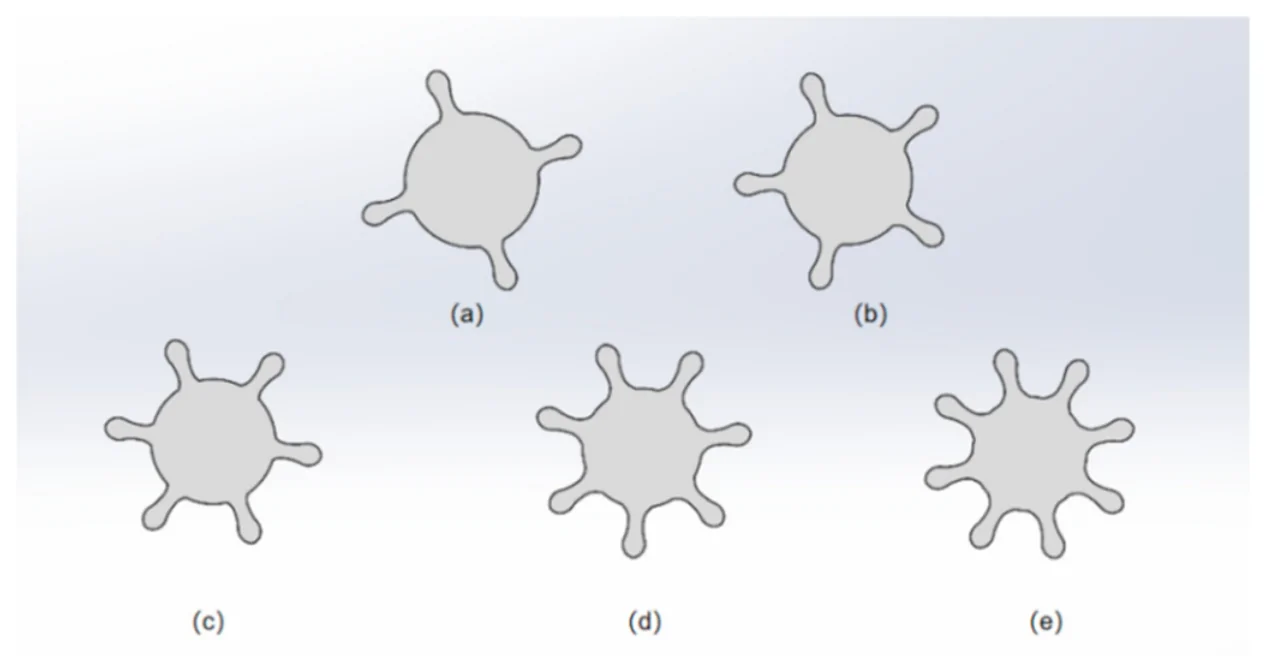
Simplified three-dimensional models of multi-lobe fibers: (**a**) four-lobe, (**b**) five-lobe, (**c**) six-lobe, (**d**) seven-lobe, (**e**) eight-lobe.

To investigate the effect of lobe number on wicking, simplified 3D fiber models with four to eight lobes were established at constant linear density (2.2 denier) and identical lobe geometry (length 5.4 μm, area 9.7 μm^2^). The core radius decreased with increasing lobe number, as shown in Figure 1: from 6.6 μm (four lobes) to 5.7 μm (eight lobes). The degree of profile irregularity (Table 1) increased from 38.3% to 46.8%, representing a 22.2% improvement for the eight-lobe design, which was therefore selected to maximize capillary wicking.

(2) Selection of the number of hollow channels

Fiber stiffness was evaluated in both axial and radial directions. For axial bending stiffness, one- to seven-hollow fiber models [20] were established at constant linear density (2 denier) and hollowness (0.2). The moment of inertia (Table 2) decreases with increasing hollow number: single-hollow shows the highest value (≈3085 μm^4^), with a sharp drop from one to two holes, relative stability from two to four, and further decline from five to seven. Thus, structures with two to four hollows offer superior bending resistance.

Radial stiffness governs resistance to lateral compression and flattening. A single-hollow cross-section, essentially a thin ring without internal support, is susceptible to deformation under load. In contrast, multi-hollow structures with interconnected ribs distribute stress and enhance compressive resistance. Finite element analysis (Table 3) shows that deformation decreases from one to four hollows, with the lowest values at three and four hollows, indicating optimal stiffness. However, deformation increases significantly at five or six hollows, suggesting that excessive hollows weaken the structure. Thus, three to four hollows offer the best radial compression performance.

Although the axial flexural rigidity slightly decreases, the gain in radial compressive stiffness is more critical for moisture wicking and anti-adhesion properties. Insufficient radial stiffness leads to collapse under compression, transforming point and line contact into area contact and compressing capillary grooves, thereby increasing adhesion while reducing wicking. The three-hollow structure reduces axial flexural rigidity by only 7.9% but decreases radial deformation by 67.9%, while also avoiding the processing difficulties associated with an excessive number of hollows. Thus, three hollows provide the best overall balance.

(3) Selection of hollow position arrangement

To improve hollow retention, the circular hollow channels were replaced with square ones, and two spatial arrangements were designed: triangular and linear. As shown in Table 4, the linear arrangement yielded a higher average moment of inertia and substantially lower average deformation than the triangular arrangement, indicating stronger axial bending stiffness and superior radial compressive stiffness. Specifically, the linear three-hollow arrangement increased the average moment of inertia by 50.8% and reduced the average deformation by 80.0% compared with the triangular arrangement. These numerical results indicate that the hollow arrangement contributes to the bending and compressive stiffness of the fiber.

Based on these results, an eight-lobe, three-hollow, in-line arrangement was selected to maximize wicking while maintaining radial compressive stiffness and simplifying spinneret fabrication. The final design is therefore an eight-lobe fiber with three square hollows in an in-line arrangement. These geometric choices are further evaluated in subsequent sections, which provide a preliminary assessment of the moisture wicking and anti-adhesion performance of the selected.

### 2.4. Numerical Simulation at Fiber-Bundle Scale

This section presents predictive bundle-scale numerical simulations. Numerical simulation has been successfully applied to predict the cross-sectional evolution of complex profiled fibers during melt spinning, with subsequent experimental validation of the predicted fiber formation [21]. This provides a methodological reference for the simulation framework adopted in this work. To evaluate the practical performance of the three-hollow eight-lobe fiber, four commercial moisture-wicking sportswear fabrics and an eight-lobe hollow fabric were selected as controls. Bundle-scale simulations were performed using a fiber bundle unit model coupled with a random cross-section simulation system. The fiber cross-sectional shapes and their corresponding linear densities are listed in Table 5.

Among these, Sportswear A–D differ in knit structure and fiber composition (e.g., Sportswear B contains polypropylene); therefore, the subsequent fabric-level comparison will focus on the three-hollow eight-lobe fabric and the eight-lobe hollow fabric, which are knitted under identical conditions. The simulations reported here are restricted to the fiber-bundle scale and serve to rank intrinsic wicking and thermal performance.

Wicking simulation. The capillary wicking effect within fiber bundles was simulated using the Fiber Bundle Unit Model (MFB) coupled with the Section Stochastic Simulation System (SG). By inputting the number of fibers and the cross-sectional area of a single fiber, the total wicking height and thermal conductivity can be computed. The model assumes that the fibers are rigid, share identical material properties, and exhibit negligible moisture absorption. Since the hollow regions do not participate in water transport, the hollow fibers were simplified into solid structures bounded by their outer contours.

The simulation parameters were set as follows: contact angle: 70°, liquid viscosity: 0.0030 Pa·s, surface tension: 0.078 N/m, liquid density: 1000 kg/m^3^, thermal conductivities of air and fiber: 0.0267 W/(m·K) and 0.2 W/(m·K), respectively, and gravitational acceleration: 9.8 m/s^2^. The fiber bundle linear density of Sportswear C (cruciform) was measured to be 76.8 dtex, and the numbers of fibers in the three-hollow eight-lobe fiber bundle and the control fiber bundles were then calculated, as shown in Table 6.

**Figure 2:**
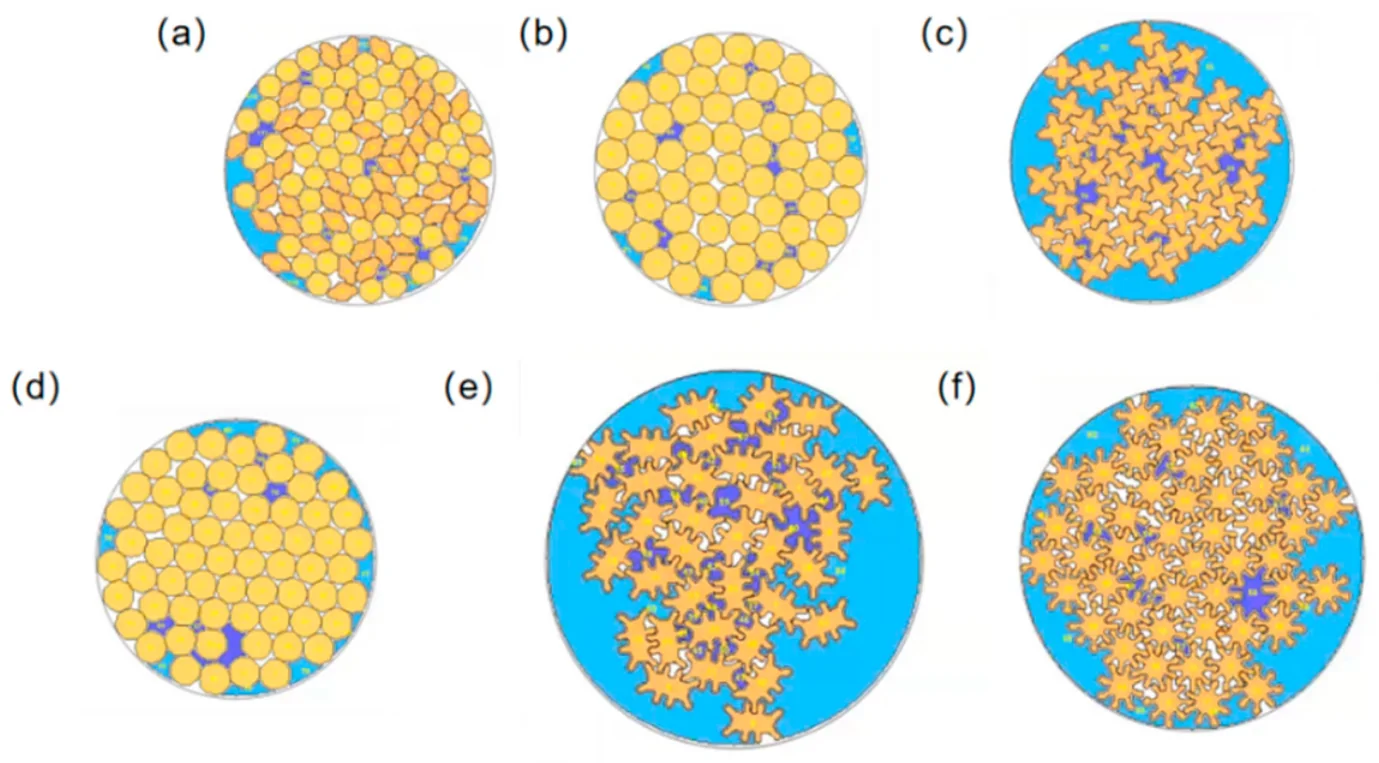
Fiber collision-shrinkage models: (**a**) 0.69 dtex/flattened cruciform + 0.77 dtex/circular; (**b**) 1.3 dtex/circular; (**c**) 1.3 dtex/cruciform; (**d**) 1.18 dtex/circular; (**e**) 1.53 dtex/eight-lobe hollow; (**f**) 2.2 dtex/three-hollow eight-lobe.

**Figure 3:**
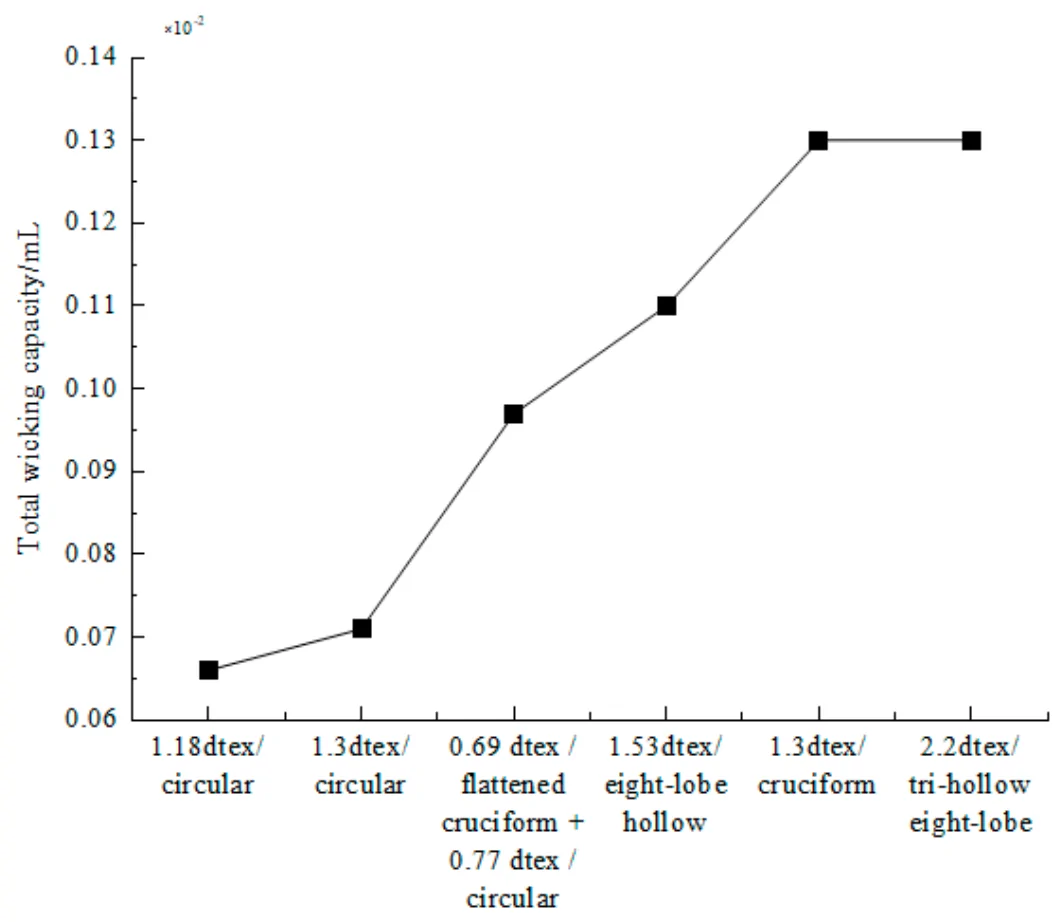
Comparison results of predicted total wicking capacity.

**Figure 4:**
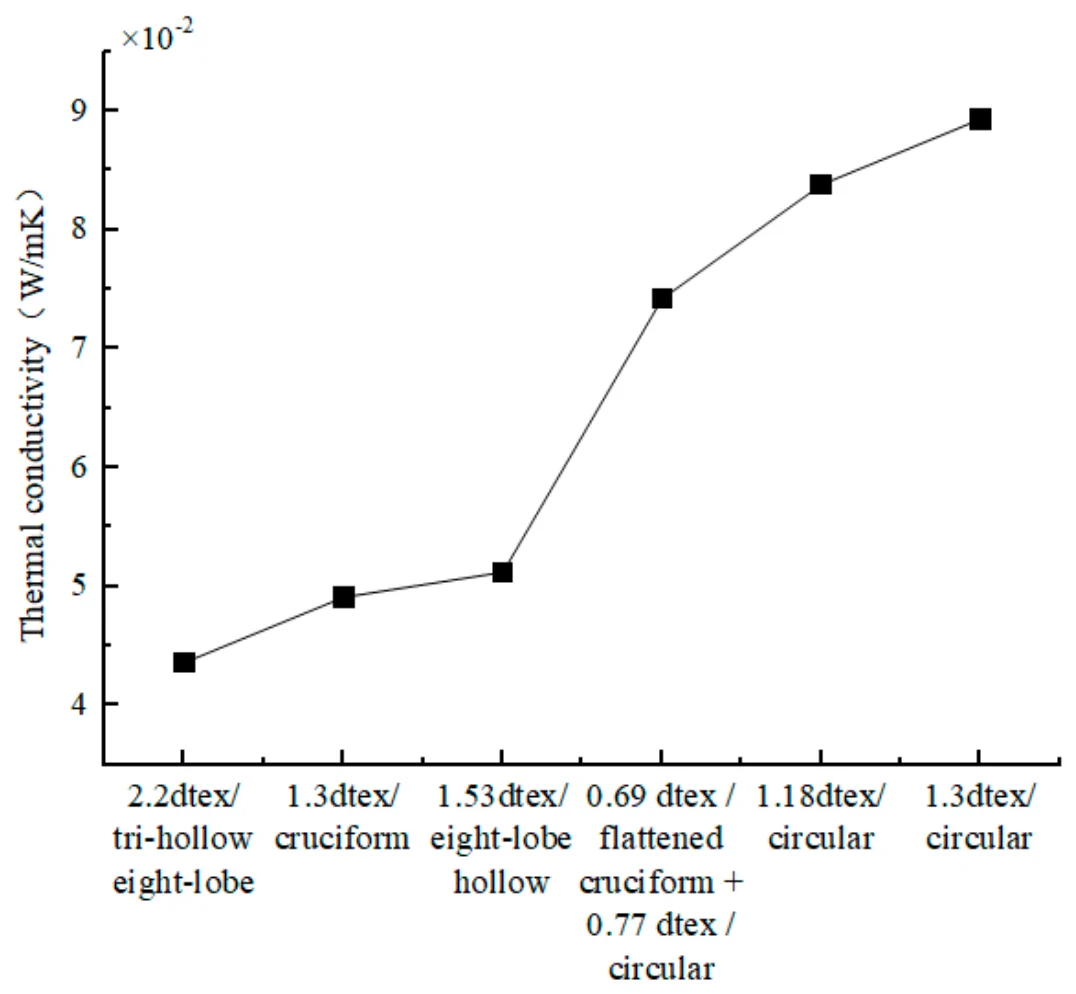
Comparison results of predicted thermal conductivity.

The number of fibers and the single-fiber linear density were input into the system to generate random cross-sectional configurations. After the configurations contracted to a stable state, the total wicking height and thermal conductivity of the fiber bundles were calculated. The simulation results include the fiber collision-shrinkage model diagrams (Figure 2), a comparison of the predicted total wicking uptake (Figure 3), and a comparison of the predicted thermal conductivity (Figure 4).

The results show that the three-hollow eight-lobe fiber bundle achieves a high level of total wicking uptake, demonstrating excellent moisture-wicking performance; concurrently, its predicted thermal conductivity is the lowest among all the cross-sections examined, suggesting good thermal insulation potential.

The radial compressive stiffness advantage of the three-hollow structure, evidenced by 67.9% lower deformation than the single-hollow structure (Table 3), directly supports the simulated wicking performance: higher radial stiffness prevents the inter-fiber capillary channels from collapsing under compression, thereby maintaining continuous water transport paths. This mechanical property also underpins anti-adhesion performance, as a more rigid cross-section resists flattening, maintains point or line contact with the skin rather than area contact, and reduces the liquid bridge force.

## 3. Spinneret Design and Melt Flow Simulation

### 3.1. Governing Equations

During the production of profiled fibers, cross-sectional formation is susceptible to multiple factors and often deviates substantially from the designed shape; theoretical guidance is therefore indispensable. Because profiled spinneret orifices are typically assembled from arranged rectangular slits, the deformation dynamics of rectangular micro-porous cross-sections provide a useful reference. Based on energy conservation, a relationship between the shape coefficient and parameters such as surface tension and extensional viscosity can be derived. By introducing a shape coefficient that is determined by the spinneret micro-hole geometry, a cross-sectional deformation dynamics model for multi-lobe fibers can be established. Under the assumption of a constant cross-sectional area along the spinning line, this model can be extended to describe the evolution of hollowness, thereby offering a theoretical basis for controlling hollow fiber formation. In extrusion flow, the polymer melt satisfies the conservation equations of mass, momentum, and energy; in particular, all continuous fluid flows must obey the mass and momentum conservation equations.

The mass conservation equation (continuity equation) is given by Equation (9), where ρ is the melt density (kg/m^3^), t refers to the time (s), and *u*, *v*, and *w* are the velocity components in the *x*, *y*, and *z* directions (m/s), respectively.

(9) $$\frac{\partial \rho }{\partial t}+\frac{\partial (\rho u)}{\partial x}+\frac{\partial \left(\rho v\right)}{\partial y}+\frac{\partial \left(\rho w\right)}{\partial z}=0$$

The momentum conservation equation (Navier–Stokes equations) states that the total momentum of a closed system remains constant in the absence of external forces [22]; the formula is given by Equation (10):

(10) $$\frac{\partial \left(\rho u_{x}\right)}{\partial t}+\nabla \cdot (\rho u_{x}\vec{u})=-\frac{\partial \rho }{\partial x}+\frac{\partial \tau_{\mathrm{xx}}}{\partial x}+\frac{\partial \tau_{\mathrm{yx}}}{\partial y}+\frac{\partial \tau_{\mathrm{zx}}}{\partial z}+\rho f_{x}\;\frac{\partial \left(\rho u_{y}\right)}{\partial t}+\nabla \cdot (\rho u_{y}\vec{u})=-\frac{\partial \rho }{\partial y}+\frac{\partial \tau_{\mathrm{xy}}}{\partial x}+\frac{\partial \tau_{\mathrm{yy}}}{\partial y}+\frac{\partial \tau_{\mathrm{zy}}}{\partial z}+\rho f_{y}\;\frac{\partial \left(\rho u_{z}\right)}{\partial t}+\nabla \cdot (\rho u_{z}\vec{u})=-\frac{\partial \rho }{\partial z}+\frac{\partial \tau_{\mathrm{xz}}}{\partial x}+\frac{\partial \tau_{\mathrm{yz}}}{\partial y}+\frac{\partial \tau_{\mathrm{zz}}}{\partial z}+\rho f_{z}$$

where *p* is the pressure on the surface of the fluid element (Pa), *τ_xx_*, *τ_yx_*, *τ_zx_*, *τ_xy_*, *τ_yy_*, *τ_zy_*, *τ_xz_*, *τ_yz_*, *τ_zz_* are the viscous stress components (Pa) acting on the element surface due to molecular viscous effects, and *f_x_*, *f_y_*, *f_z_* are the body forces per unit mass in the *x*, *y*, *z* directions, respectively (m/s^2^).

The energy conservation equation states that the total energy within a system remains constant. Its equation is given by Equation (11):

(11) $$\frac{\partial \left(\rho E\right)}{\partial t}+\nabla \cdot \left[\vec{u}\left(\rho E+p\right)\right]=\nabla \cdot \left[k_{\mathrm{eff}}-\sum_{j}\nabla T-\sum_{j}h_{j}J_{j}+\left(\tau_{\mathrm{eff}}\cdot \vec{u}\right)\right]+S_{h}$$

where *E* is the total energy per unit mass of the fluid element (J/kg), h is the enthalpy (J/kg), $h_{j}$ is the enthalpy of component j, $k_{\mathrm{eff}}$ and $\tau_{\mathrm{eff}}$ are the effective thermal conductivity and the turbulent thermal conductivity, respectively (W/(m·K)), $J_{j}$ is the diffusion flux of component j, and $S_{h}$ is a user-defined volumetric heat source term. The constitutive equation is a mathematical model that describes the macroscopic mechanical response of a material. In classical continuum mechanics, it takes the form of an equation relating the deviatoric stress tensor to the rate-of-deformation tensor for viscoelastic fluid flow. Constitutive models for polymer melt flow are generally classified into generalized Newtonian flow models and viscoelastic constitutive models [23].

The PET melt is modeled as a generalized Newtonian fluid. Its shear-rate-dependent viscosity is characterized by the Bird–Carreau model (Equation (12)),

(12) $$\eta =\eta_{\infty }+\left(\eta_{0}-\eta_{\infty }\right){\left(1+\lambda^{2}\cdot \gamma^{2}\right)}^{\frac{\left(n-1\right)}{2}}$$

where *η* is the viscosity (Pa·s), *η*_0_ is the zero-shear viscosity (Pa·s), $\eta_{\infty }$ is the infinite-shear viscosity (Pa·s), *γ* is the shear rate (s^−1^), *λ* is the relaxation time (s), and n is the non-Newtonian index. A profiled spinneret orifice can be approximated as an assembly of multiple rectangular slits. For a single rectangular slit, the reduction in surface area of the extruded melt is balanced by viscous dissipation, leading to an evolution equation for the shape factor *k* [24], as given in Equation (13):

(13) $$\frac{dk}{\left(k^{2}-1\right)}=-\frac{F_{s}}{2\eta \sqrt{A}V}dz$$

Here, $F_{s}$ is the surface tension, *A* the cross-sectional area (m^2^), *V* the flow velocity (m/s), and *η* the extensional viscosity (Pa·s). This equation describes how the cross-section tends to circularize under surface tension as the melt travels down the spinline.

For a multi-lobe spinneret orifice, the deformation dynamics are modified because the melt exits from multiple interconnected slits. The complex geometry introduces a geometric correction factor *ξ* that accounts for the deviation from an ideal single-slit behavior. *ξ* is not an adjustable fitting parameter; it is determined from the actual micro-hole geometry using finite element pre-processing. Specifically, *ξ* is computed as the ratio of the actual rate of shape change (obtained from a preliminary simulation of the multi-slit geometry) to that predicted by Equation (14) for a single slit with the same hydraulic diameter. Thus, *ξ* depends on measurable or computable design parameters: the slit width, the number of lobes, the gap size between slits, and the arrangement (triangular vs. linear). With this correction, the deformation model for a multi-lobe fiber becomes [25]:

(14) $$\xi \frac{dk}{\left(k^{2}-1\right)}=-\frac{F_{s}}{2\eta \sqrt{A}v}dz$$

Assuming that the cross-sectional area of a small hollow fiber segment remains constant along the spinline (mass conservation), and considering a circular lamina of arbitrary radius $r_{\left(i\right)}$ located between the inner and outer walls, the radius changes from $r_{\left(i\right)}$ to $r_{\left(i+1\right)}$. after passing through a small distance. The hollowness $B_{\left(i\right)}$ (cavity area fraction) then evolves as Equation (15):

(15) $$B_{\left(i\right)}=\frac{\pi {\left(R_{i}+\Delta R_{i}\right)}^{2}}{A_{i}+\pi {\left(R_{i}+\Delta R_{i}\right)}^{2}}$$

where $R_{i}$ is the outer radius at step *i*, $\Delta R_{i}$ the radial change due to drawing, and $A_{i}$ the cross-sectional area of the solid polymer (excluding the cavity). This equation allows the prediction of hollowness along the spinline and is used to guide the cooling conditions to prevent cavity closure.

### 3.2. Micro-Hole Design and Stress Analysis

Based on the general requirements for profiled spinneret micro-orifice design, a slit width of 0.05 mm was preliminarily selected. To determine the gap size between slits and the number of unit divisions for the complex geometry, three-dimensional modelling and static stress analysis were performed for different design schemes. By combining the number of unit divisions with the gap size, the following four groups were subjected to stress testing:

(a) number of units: 1, number of gaps: 3, gap size: 0.06 mm;
(b) number of units: 1, number of gaps: 3, gap size: 0.08 mm;
(c) number of units: 2, number of gaps: 4, gap size: 0.06 mm;
(d) number of units: 3, number of gaps: 5, gap size: 0.06 mm.

**Figure 5:**
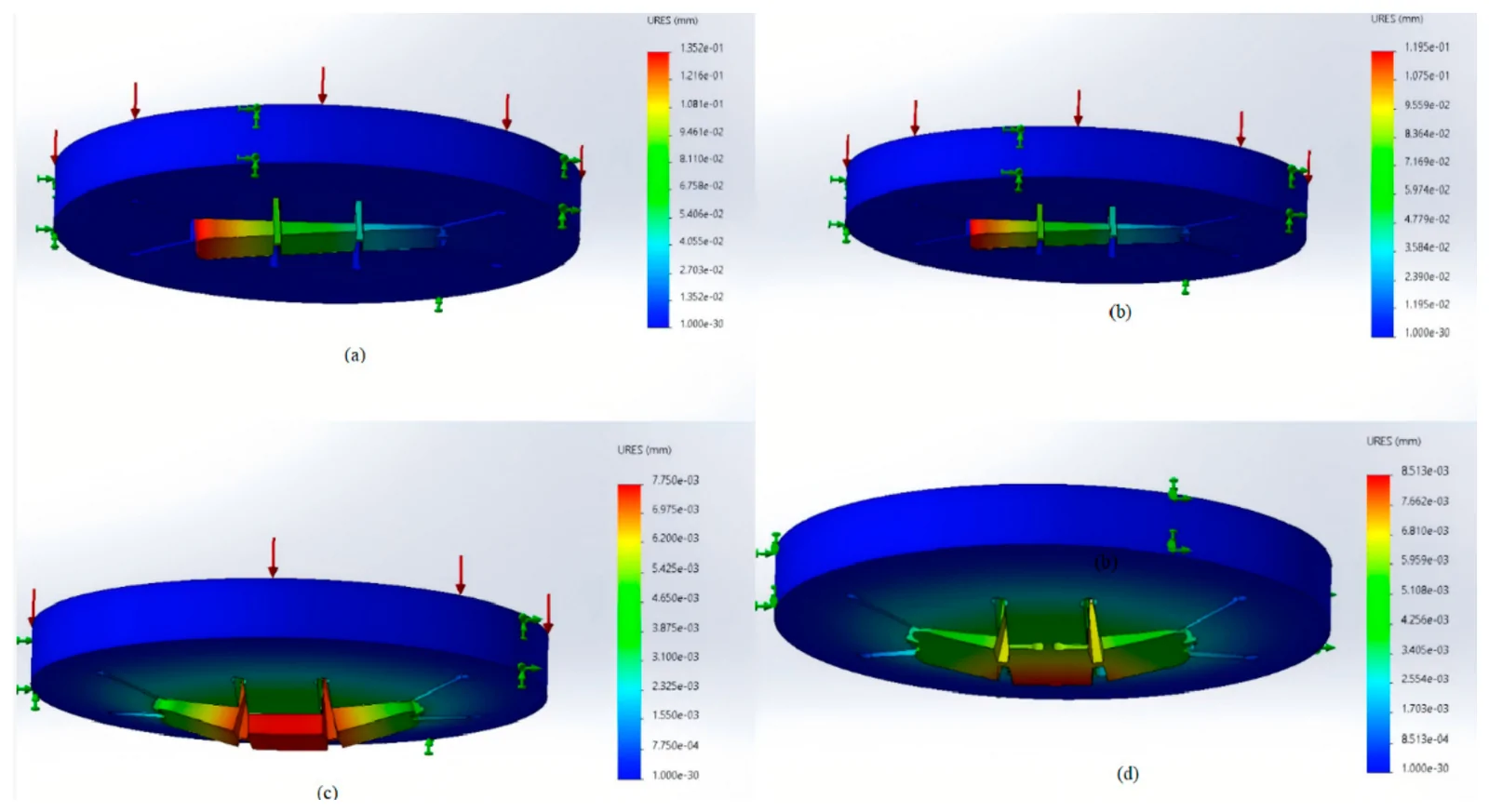
Total deformation results of the static structural analysis: (**a**) 3 gaps, gap size 0.06 mm; (**b**) 3 gaps, gap size 0.08 mm; (**c**) 4 gaps, gap size 0.06 mm; (**d**) 5 gaps, gap size 0.06 mm. The material was a AISI316 stainless-steel plate, and an inlet pressure of 20 MPa was applied.

The material used was an AISI 316 stainless-steel plate(Taiyuan Iron & Steel (Group) Co., Ltd., Taiyuan, China). A pressure of 20 MPa was applied to the inlet face, exceeding the typical spinning pressure to provide a safety margin. The deformation results and the maximum deformation are shown in Figure 5 and Table 7, respectively.

Stress analysis reveals that a single integral unit, supported at only one point, tilts under pressure. Even with a gap size of 0.08 mm, deformation remains considerable, confirming that the single-unit design cannot effectively resist pressure. In contrast, increasing the number of profiled units to two or three introduces additional support points, resulting in a marked decrease in deformation. Despite a marginally higher deformation than the two-unit configuration, the three-unit design is structurally preferable because it provides better load distribution and processing feasibility, with deformation remaining well within the empirical tolerance and satisfying all spinning strength requirements. Accordingly, the final design adopts a slit width of 0.05 mm, three unit divisions, and a slit gap of 0.06 mm.

### 3.3. Formulations and Numerical Simulations 

In the numerical simulation of melt flow, the following assumptions are made: (a) The melt is a non-isothermal, incompressible, non-Newtonian fluid, (b) A no-slip condition is applied at the spinneret orifice wall, and (c) The effects of gravity and inertial forces are neglected.

#### 3.3.1. Numerical Simulation of Spinning Formation

A geometric model of the three-hollow eight-lobe fiber is established for the portion extruded from the spinneret orifice, comprising two flow regions: the flow domain of the PET melt inside the spinneret channel (0.4 mm) and the domain where the melt is extruded and then solidified into the fiber (40 mm). Under the stated assumptions, the mass conservation equation reduces to the divergence-free condition for velocity, and the momentum and energy conservation equations are simplified accordingly, as given in Equations (16)–(18):

(16) ∇·V=0

(17) $$\rho \frac{dV}{dt}=-\nabla p+\nabla \tau$$

(18) $$\rho C_{p}=\frac{dT}{dt}=-\nabla q+\frac{\tau }{\nabla v}$$

where *∇* is the del operator; *V* is the velocity vector (m/s); *τ* is the stress tensor; *ρ* is the density (g/cm^3^); *p* is the pressure (Pa); *T* is the melt temperature (K); $C_{p}$ is the specific heat at constant pressure (J/(kg·K)); and *q* is the heat flux (W/m^2^).

The rheological parameters of the PET material were as follows: non-Newtonian index: *n* = 0.55, relaxation time: λ=0.20 s, zero-shear viscosity: $\eta_{0}$ = 271.45 Pa·s, infinite-shear viscosity: $\eta_{\infty }$ = 33.78 Pa·s, density: ρ = 1.27 g/cm^3^, thermal conductivity: 0.21 W/(m·K), and specific heat capacity: $C_{p}$ = 3.45 kJ/(kg·K).

The three-dimensional melt-flow simulation was performed. The governing equations were solved using the finite element method, and the Bird–Carreau constitutive equation was selected. The flow conditions were set as follows: at the inlet face, a prescribed flow rate per single orifice was applied; a no-slip boundary condition (both tangential and normal velocities set to zero) was applied on the spinneret wall; the free surface corresponded to the extruded melt surface; and an axial draw velocity was imposed at the outlet face. The thermal boundary conditions included an initial quenching height of 20 mm, a quenching air speed of 1.76 m/s, and a quenching air temperature of 45.2 °C.

**Figure 6:**
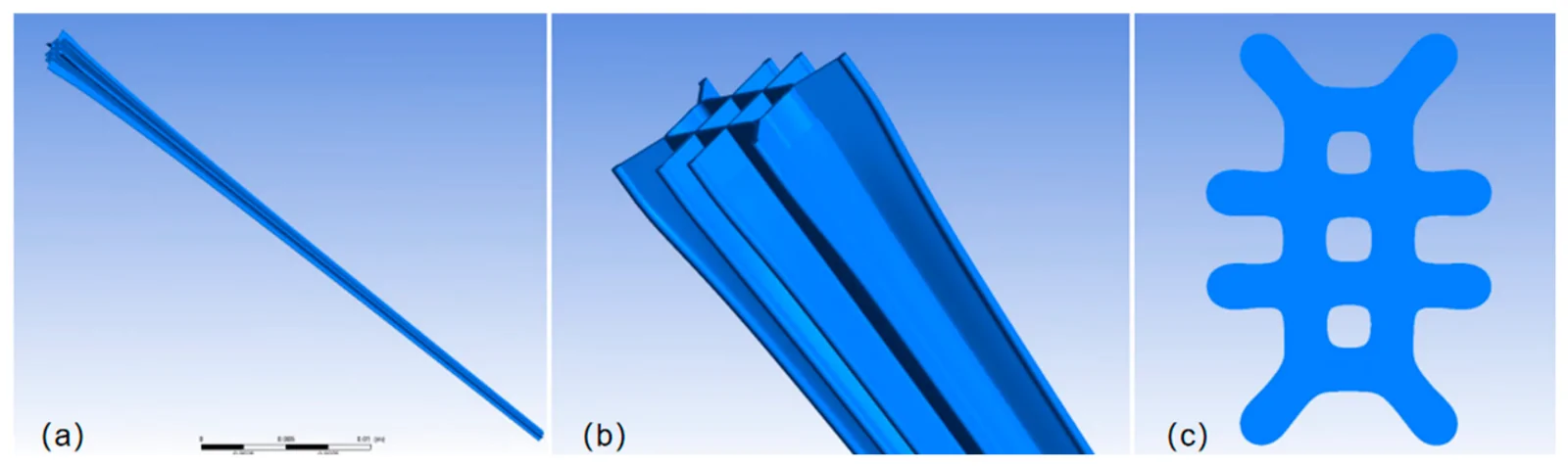
Numerical simulation of extrusion shape for tri-hollow eight-lobe fiber: (**a**,**b**) extrusion geometric model; (**c**) cross-sectional shape at the end of the extrusion section. Simulation conditions: PET melt, Bird–Carreau model, quenching air speed 1.76 m/s, initial quenching height 20 mm, air temperature 45.2 °C.

Figure 6 shows the numerically simulated extrusion shape of the three-hollow eight-lobe fiber. The simulation results indicate that when the melt is extruded, it first undergoes a certain degree of die swell, and then the cross-sectional deformation gradually stabilizes under the axial draw velocity. At approximately 40 mm from the spinneret surface, the extrusion forming process stabilizes, the cross-sectional shape is basically fixed, and a well-defined three-hollow eight-lobe structure is formed, confirming the feasibility of the design.

#### 3.3.2. Numerical Simulation of Shear Rate, Velocity, and Pressure

**Figure 7:**
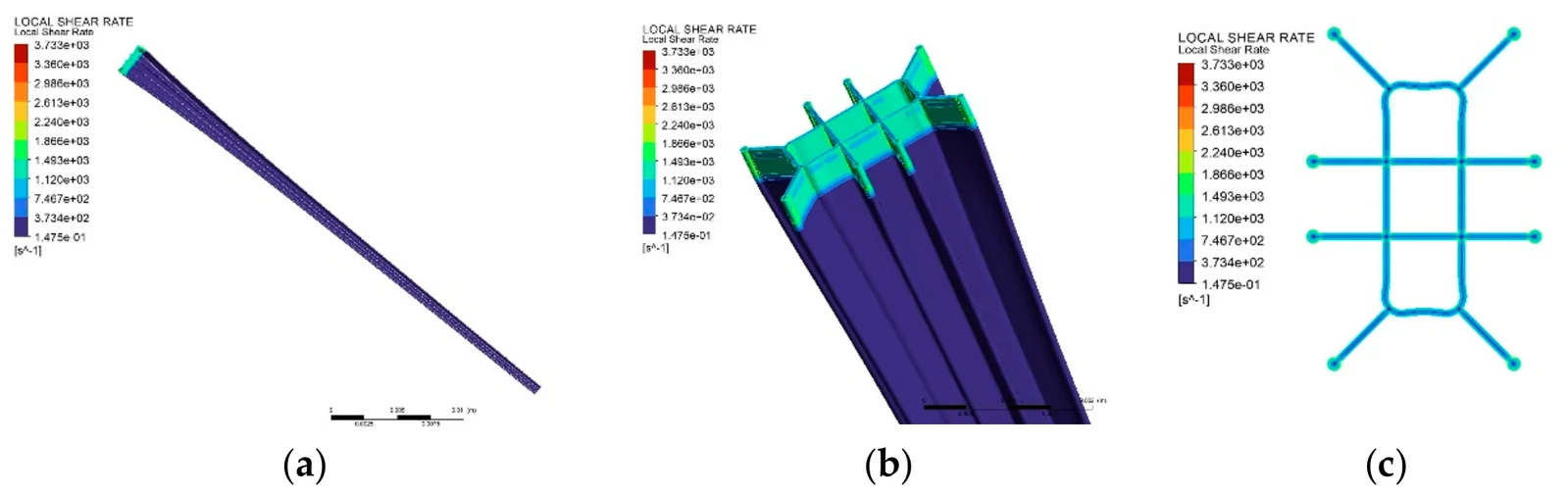
Shear rate distribution: (**a**,**b**) overall shear rate distribution; (**c**) shear rate distribution at the initial cross-section of the extrusion section.

The shear rate distribution is shown in Figure 7. The simulation results indicate that the shear rate decreases in the axial direction as the melt moves away from the inlet face. Within the spinneret micro-orifice channel, the maximum shear rate occurs at the channel wall and is distributed symmetrically with respect to the symmetry plane of each complex profiled unit. The maximum shear rate is 3733 s^−1^, which falls within the typical melt spinning shear rate range of 2500–10,000 s^−1^, indicating that the melt flow behaviour in the channel is appropriate: the shear is neither so high as to cause excessive molecular chain scission nor so low as to compromise flow stability.

**Figure 8:**
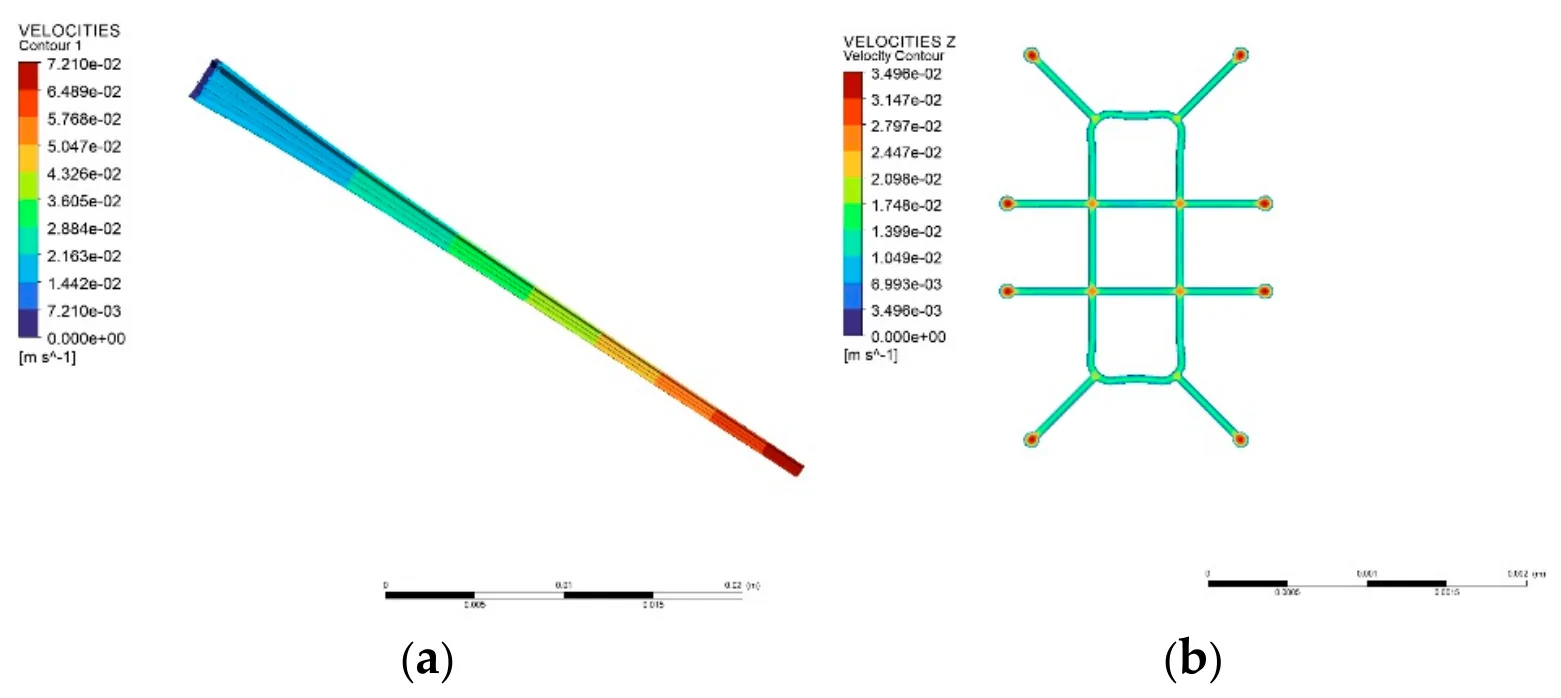
Velocity distribution: (**a**) overall velocity distribution; (**b**) velocity distribution at the initial cross-section of the extrusion section.

The velocity distribution is shown in Figure 8. The overall velocity distribution indicates that the velocity increases in the axial direction as the melt moves away from the inlet face. The cross-sectional velocity distribution shows that the maximum velocity occurs in the region of the lobe tip centers and the junction nodes. The maximum velocity at the initial cross-section of the extrusion section is 0.035 m/s. The characteristics of the velocity distribution correspond well with the cross-sectional geometry, which is beneficial for preserving the fiber cross-sectional shape.

**Figure 9:**
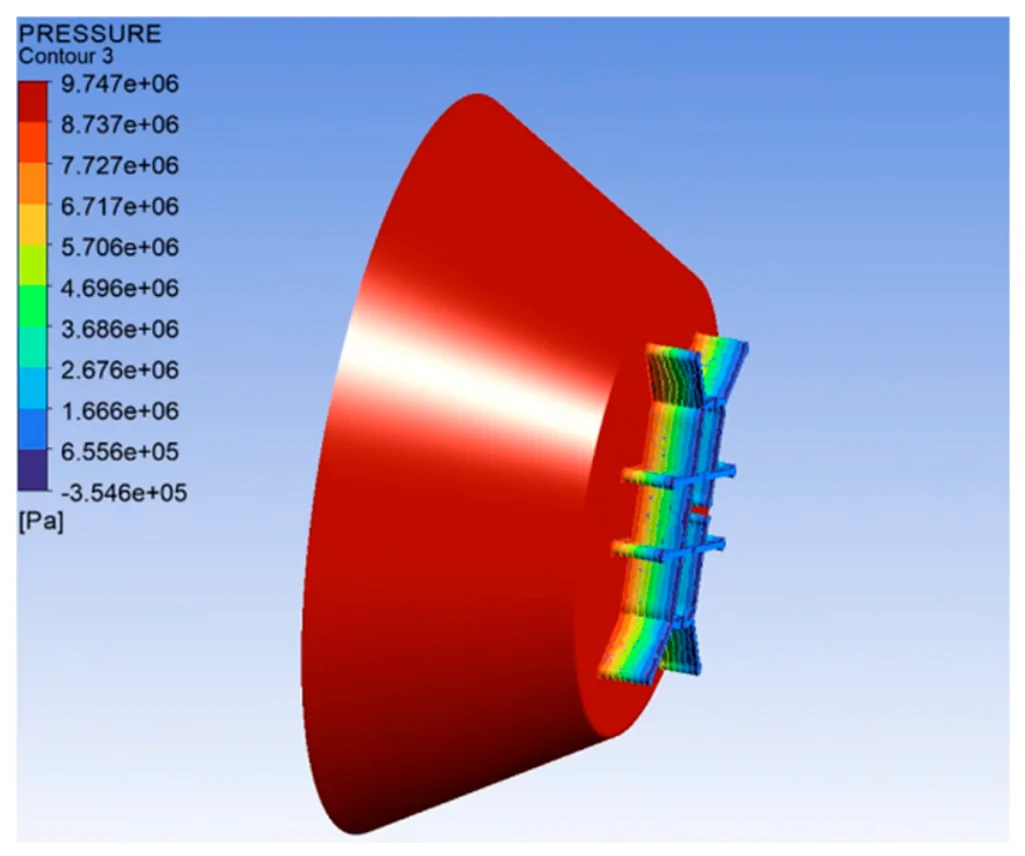
Pressure distribution.

The pressure distribution is shown in Figure 9. The numerical simulation shows that the pressure decreases along the channel axis, and the magnitude of pressure variation begins to increase near the micro-orifice section. The maximum pressure is 9.75 MPa, which falls within the typical working pressure range for conventional PET melt spinning, validating the rationality of the spinneret orifice design and indicating that under the given process conditions, the maximum pressure remained below the equipment limit, and no melt leakage was observed.

#### 3.3.3. Mesh Resolution and Convergence Validation

**Figure 10:**
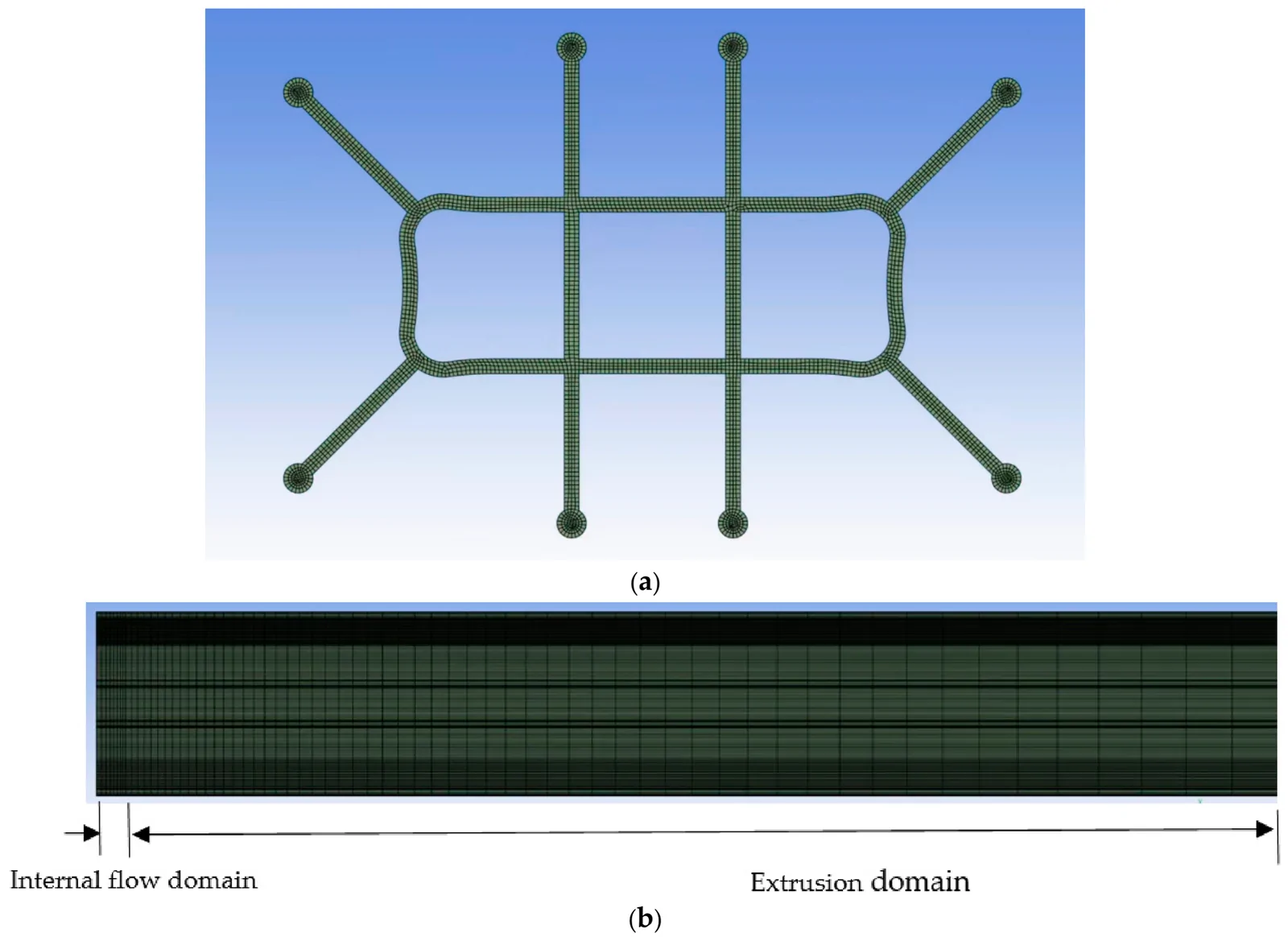
Meshing of the three-hollow eight-lobe geometric model: (**a**) cross-sectional mesh; (**b**) extrusion direction mesh.

The geometric model was discretized using quadrilateral elements. The mesh was generated by partitioning the cross-section and sweeping along the extrusion direction. The computational domain comprises the internal channel (0.4 mm) and the extrusion region (40 mm). Mesh refinement was applied near the spinneret outlet to improve convergence [26]. The cross-sectional and extrusion-direction mesh distributions are shown in Figure 10.

A mesh independence study was conducted to verify that the simulation results are independent of mesh density. Three mesh density levels were designed: the cross-sectional domain was divided into 5, 6, and 9 partitions, and the extrusion-direction domain was divided into 75, 100, and 1 segments, respectively. These configurations are denoted as Models a, b, and c. The corresponding partition numbers and total element counts are summarized in Table 8.

**Figure 11:**
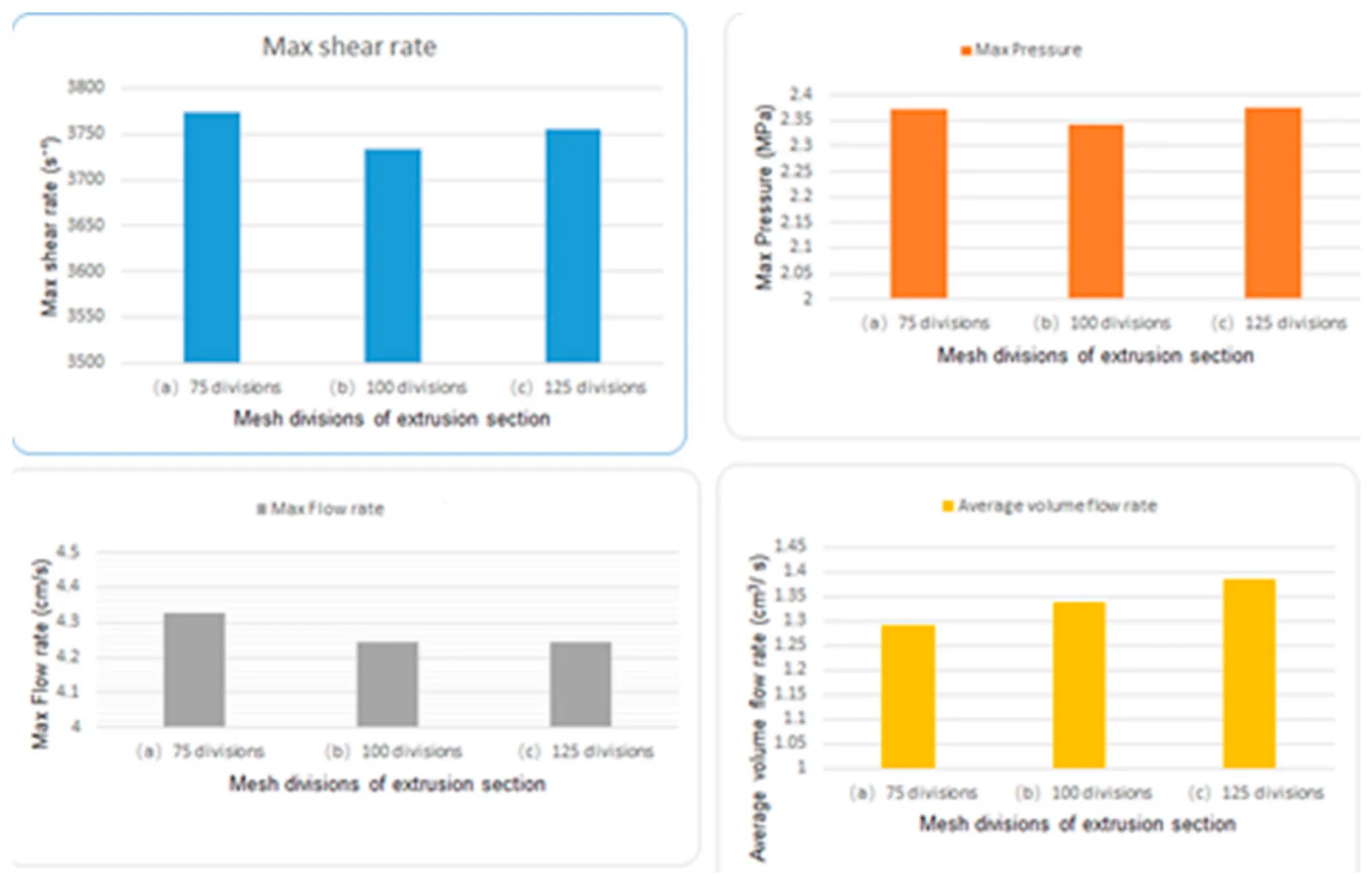
Comparison of key flow parameters for three mesh densities: maximum shear rate; maximum pressure; maximum flow velocities; average volume velocities.

All simulations were performed under identical conditions (material properties, boundary conditions, and solver settings) except for mesh density. The following key flow parameters were extracted for comparison: maximum shear rate, maximum pressure, maximum velocity, and average volume velocities. The results are presented in Figure 11.

The comparison shows that the relative differences in all flow parameters among the three mesh densities are within acceptable limits (<3%). This indicates that once the mesh quality meets the basic requirement, further refinement does not significantly affect the flow field results, while it substantially increases computational cost. Therefore, considering both accuracy and efficiency, Model b (6 cross-sectional partitions, 100 extrusion direction segments, 388,648 elements) was adopted for all production simulations in this study.

#### 3.3.4. Numerical Simulation of Extrusion Swell and Bonding

**Figure 12:**
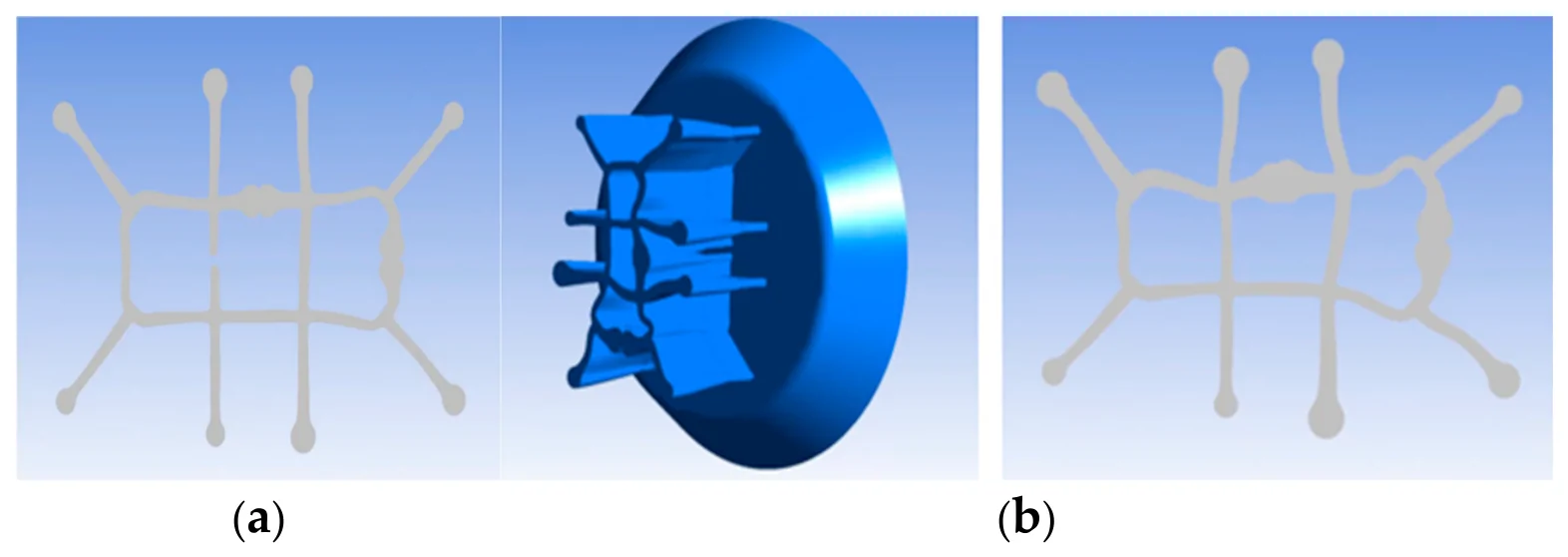
(**a**) Pressure distribution extrusion swell at a distance of 0.2 mm from the spinneret outlet. (**b**) Extrusion swell phenomenon at a distance of 0.5 mm from the spinneret outlet.

Whether the melt can bond successfully after extrusion to form an intact hollow structure is crucial for verifying the feasibility of the design. The simulation setup followed the melt flow model described in Section 3.1 and Section 3.2, using the Bird–Carreau constitutive equation and the boundary conditions listed in Section 3.3.1. The extrusion swell and inter-lobe bonding process is illustrated in Figure 12.

At 0.2 mm from the spinneret outlet (Figure 12a), the gap between each unit has narrowed markedly, but bonding is not yet complete. At 0.5 mm (Figure 12b), the melt has achieved bonding through die swell. Judging from the cross-sectional shape at 2 mm, a well-defined three-hollow structure has formed. These observations confirm that the micro-hole design enables successful inter-lobe coalescence and spinnability under the given conditions. To further clarify the underlying mechanism, a quantitative analysis of viscoelastic relaxation, surface tension, and local cooling rate was performed.

First, the viscoelastic nature of the PET melt governs the extent of die swell. The relative importance of elastic to viscous effects is characterized by the Deborah number [27], defined as:

(19) $$\mathrm{De}=\lambda /t_{f}$$

where *λ* = 0.20 s is the relaxation time and $t_{f}$ is the characteristic flow time. Taking the coalescence distance *L* = 0.5 mm and the extrusion velocity *V* ≈ 0.035 m/s (as obtained from the velocity field simulation), $t_{f}=L/V\approx$ 0.014 s, giving *De* ≈ 14. Since *De* > 1, elastic stresses prevail over viscous stresses right after the melt exits the spinneret, producing substantial die swell that forces the initially separated lobes together; this contact is an essential precondition for their subsequent coalescence.

Once the lobe surfaces are in proximity, surface tension acts to merge them. The competition between viscous and capillary forces is expressed by the capillary number [28]:

(20) $$Ca=\eta_{0}V/\gamma$$

where $\eta_{0}$ = 271.45 Pa·s is the zero-shear viscosity and *γ* is the surface tension of the PET melt (taken as 0.03 N·m^−1^ from the literature). Substitution yields Ca ≈ 317, implying that viscous forces dominate over surface tension at the macroscopic scale. However, in the confined gap between approaching lobes, the inter-lobe spacing decreases to approximately 20 μm, as indicated by the simulated geometry at 0.2 mm from the outlet (Figure 10a). When a liquid bridge forms between the two surfaces, its meniscus curvature radius can be approximated as half the gap width. Under this small radius, the Laplace pressure difference Δp=2γ/r becomes significant [29], reaching approximately 6 kPa. This local pressure gradient actively drives melt flow into the gap, completing the bonding process within a very short distance. By adopting the minimum expected radius (10 μm rather than a larger value), the estimated driving force represents a conservative upper bound, confirming that surface tension is sufficient to ensure coalescence even in the most critical stage.

The role of cooling is equally critical. The initial quenching height is set at 20 mm, so the coalescence zone (0–0.5 mm from the spinneret) lies well within the region unaffected by forced convective cooling. The axial temperature profile extracted from the simulation shows a temperature drop of less than 2 K over this 0.5 mm distance. With the characteristic flow time $t_{f}\approx$ 0.014 s, the local cooling rate can be estimated as [30] $\dot{T}\equiv \Delta T/t_{f}\approx 2\;K/$0.014 s ≈ 140 K/s. To evaluate the consequent viscosity change, an Arrhenius-type viscosity–temperature relationship is employed [31]:

(21) $$\eta \left(T\right)=\eta_{0}exp\left[\frac{E}{R}\left(\frac{1}{T}-\frac{1}{T_{0}}\right)\right]$$

where the flow activation energy of PET is approximately 41.6 kJ/mol, corresponding to E/R≈ 5000 K, consistent with reported values for PET melts [27]. Taking $T_{0}=$ 287 °C = 560 K and *T* = 558 K (corresponding to the maximum temperature drop of 2 K in the coalescence zone), the melt viscosity increases from $\eta_{0}$ = 271.45 Pa·s to approximately 280 Pa·s, corresponding to a relative increase of about 3.3%. This negligible rise (well below 5%) confirms that the low-viscosity condition favorable for rapid coalescence is preserved throughout the bonding region. In contrast, if cooling were initiated earlier (i.e., with a smaller initial quenching height), the melt would enter the forced cooling zone before coalescence is complete, leading to a higher cooling rate, a sharp viscosity increase, and hindered bonding.

In summary, the successful bonding of the three-hollow eight-lobe fiber arises from a favorable sequence: elastic-driven die swell (*De* ≈ 14) ensures initial lobe contact, surface-tension-induced Laplace pressure (Δp≈ 6 kPa, based on a liquid-bridge curvature of r∼10 μm derived from the 20 μm inter-lobe gap) completes local coalescence, and the deferred cooling maintains a nearly constant low viscosity (increase from 271.45 Pa·s to 280 Pa·s) throughout the critical coalescence window. These quantitative insights substantiate the simulation results and verify the spinnability of the designed micro-hole geometry.

## 4. Preparation and Mechanical Test

During melt spinning, the extruded high-temperature melt is easily affected by surface tension and cooling conditions, which may cause cross-sectional deformation or hollow closure. Therefore, precisely regulating the cooling process to preserve the cross-sectional profile is the key challenge for achieving controllable preparation. In this section, PET melt spinning experiments are carried out to investigate the effects of initial quenching height and quenching air velocity on the cross-sectional irregularity and hollowness of as-spun fibers. Optimal process parameters are determined by balancing cross-sectional fidelity and spinnability, and the mechanical properties of the resulting fibers are tested to ensure a supply of qualified raw material for subsequent fabric weaving.

### 4.1. Spinning Process and Raw Materials

The spinning experimental equipment includes a multi-component melt spinning machine, a quenching device, a winding unit, and a drying oven. The raw material for spinning is ordinary bright polyester (PET) chips. The rheological parameters of the PET are as follows: non-Newtonian index: 0.55, relaxation time: 0.20 s, zero-shear viscosity: 271.45 Pa·s, infinite-shear viscosity: 33.78 Pa·s, and density: 1.27 g/cm^3^. To prevent moisture in the PET chips from inducing polymer hydrolysis, viscosity reduction, and spinnability degradation, the PET chips must be vacuum-dried in a dryer for 10 h according to a preset program. The spinning process route is as follows: the dried PET chips are conveyed, heated, melted, and extruded through a screw extruder. The temperatures of the six zones of the screw extruder are set at 270 °C (Zone 1), 280 °C (Zone 2), and 290 °C (Zones 3–6), and the spinning beam temperature is set at 287 °C.

### 4.2. Effect of Initial Quenching Height on Fiber Cross-Sectional Formation

With the single-hole volumetric flow rate, spinning temperature, and winding speed held constant, the cooling condition is the most significant factor influencing fiber cross-sectional formation. To investigate the effect of initial quenching height, comparative experiments were conducted at initial quenching heights of 40 mm and 20 mm, while the winding speed was 2800 m/min and the quenching air velocity was fixed at 4.6 m/s, the quenching length at 150 mm, and the air temperature at 45.2 °C.

**Figure 13:**
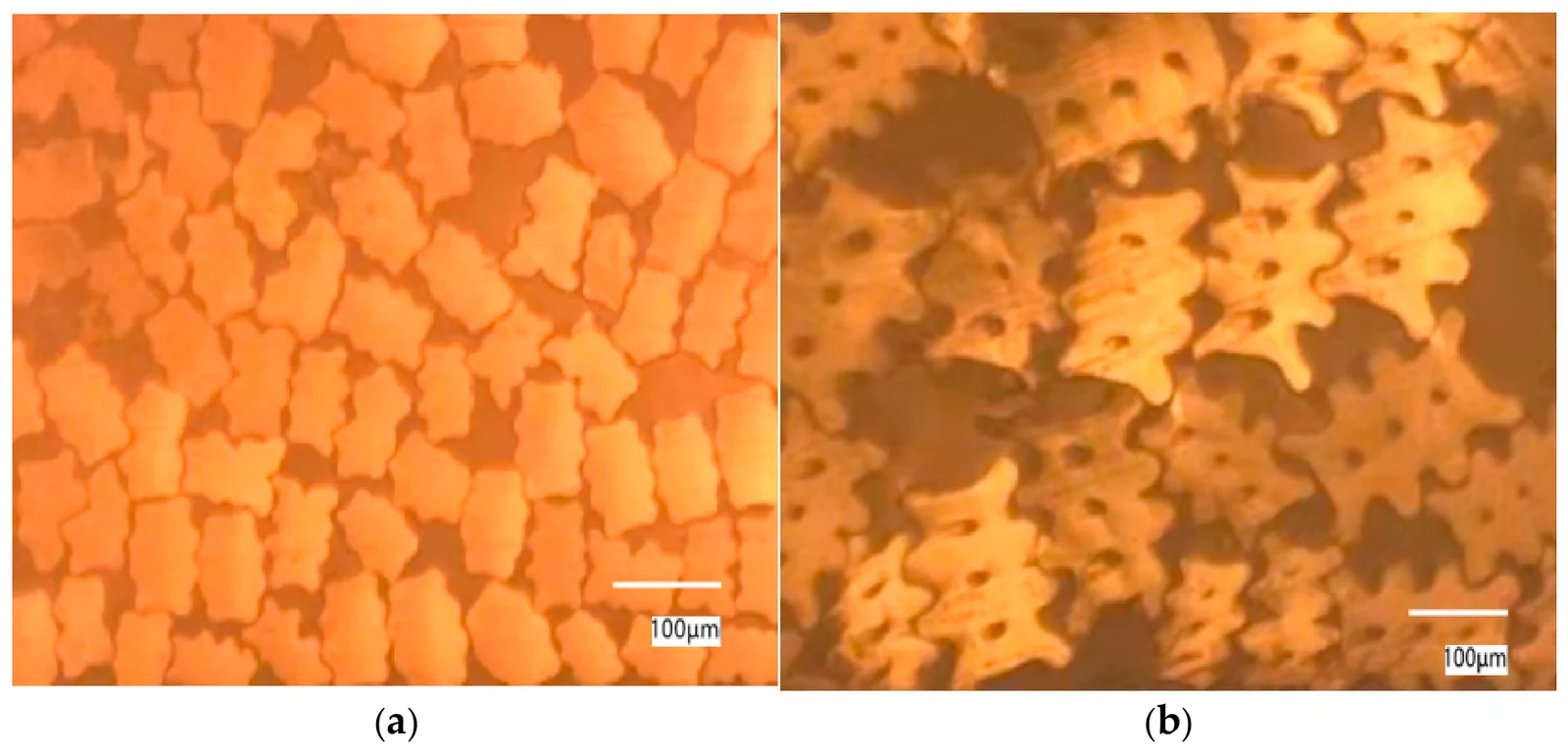
As-spun cross sections of the three-hollow eight-lobe fiber at initial blowing heights of (**a**) 40 mm and (**b**) 20 mm. The winding speed was 2800 m/min, the quenching air velocity was 4.6 m/s, the quenching length was 150 mm, and the air temperature was 45.2 °C. Scale bars: 100 μm.

The experimental results are shown in Figure 13. It is obvious that the initial quenching height has a pronounced effect on the cross-sectional formation of tri-hollow eight-lobe fibers. At an initial quenching height of 40 mm, the fiber cross-section failed to develop a distinct hollow structure, and the outer contour was rather blurred. When the initial quenching height was reduced to 20 mm, the fiber cross-section exhibited a clear hollow structure, with a sharply defined eight-lobe outer contour.

The mechanism underlying this phenomenon is as follows. Upon exiting the spinneret, the melt undergoes extensional flow that induces a non-circular, profiled deformation matching the micro-hole geometry; simultaneously, surface tension drives the cross-section toward a minimum-surface-area (circular) shape, thereby acting against the flow-induced profiled feature. The initial quenching height directly determines how long the extruded melt remains under low-viscosity conditions before significant cooling begins. A higher quenching start height prolongs this high-temperature, low-viscosity residence, giving surface tension sufficient time to drive the melt toward circularity, which erases both the flow-induced deviation from roundness and the nascent hollow structure. Conversely, a lower quenching start height brings the melt into contact with the cooling air flow earlier, leading to a rapid increase in viscosity. This sharp viscosity rise effectively suppresses the surface-tension-driven shape relaxation and freezes the flow-induced profiled cross-section together with the hollow interior generated by the spinneret design. Therefore, to achieve better cross-sectional fidelity by favoring flow-induced shaping and minimizing surface-tension-induced closure, the initial blowing height was set at 20 mm.

### 4.3. Effect of Cooling Air Velocity on Fiber Cross-Section Formation

**Figure 14:**
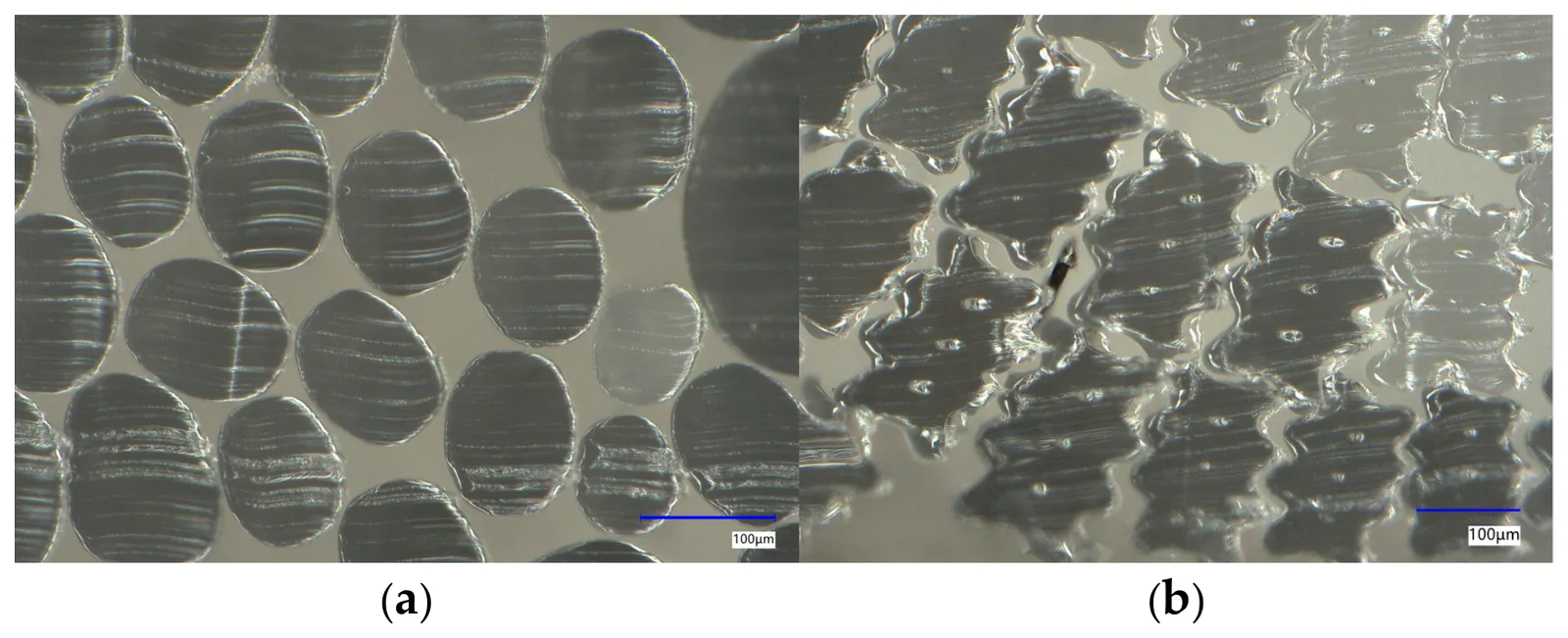
As-spun cross sections of the three-hollow eight-lobe fiber at a blowing speed of (**a**) 0 m/s and (**b**) 1.76 m/s. The initial quenching height was 20 mm, the quenching length was 150 mm, and the quenching air temperature was 45.2 °C. Scale bars: 100 μm.

**Figure 15:**
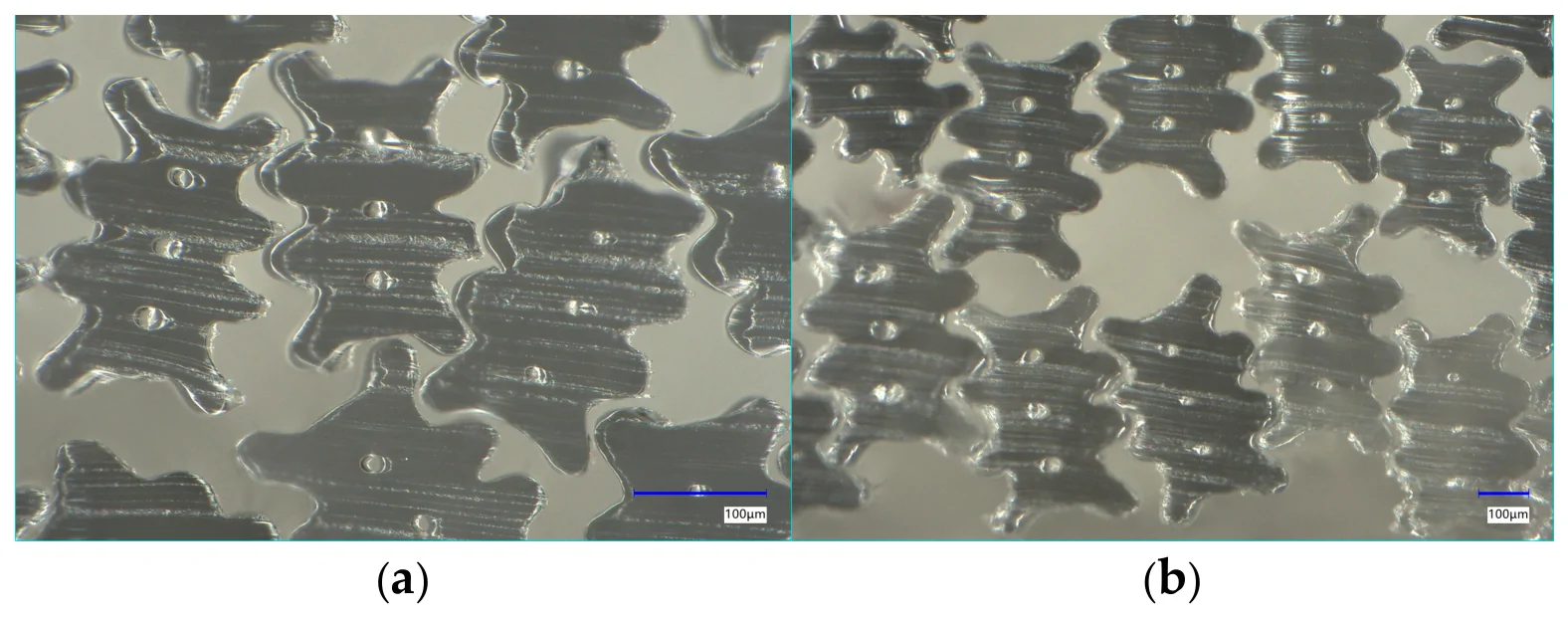
As-spun cross sections of the three-hollow eight-lobe fiber at a blowing speed of (**a**) 2.35 m/s and (**b**) 3.40 m/s. The initial quenching height was 20 mm, the quenching length was 150 mm, and the quenching air temperature was 45.2 °C. Scale bars: 100 μm.

**Figure 16:**
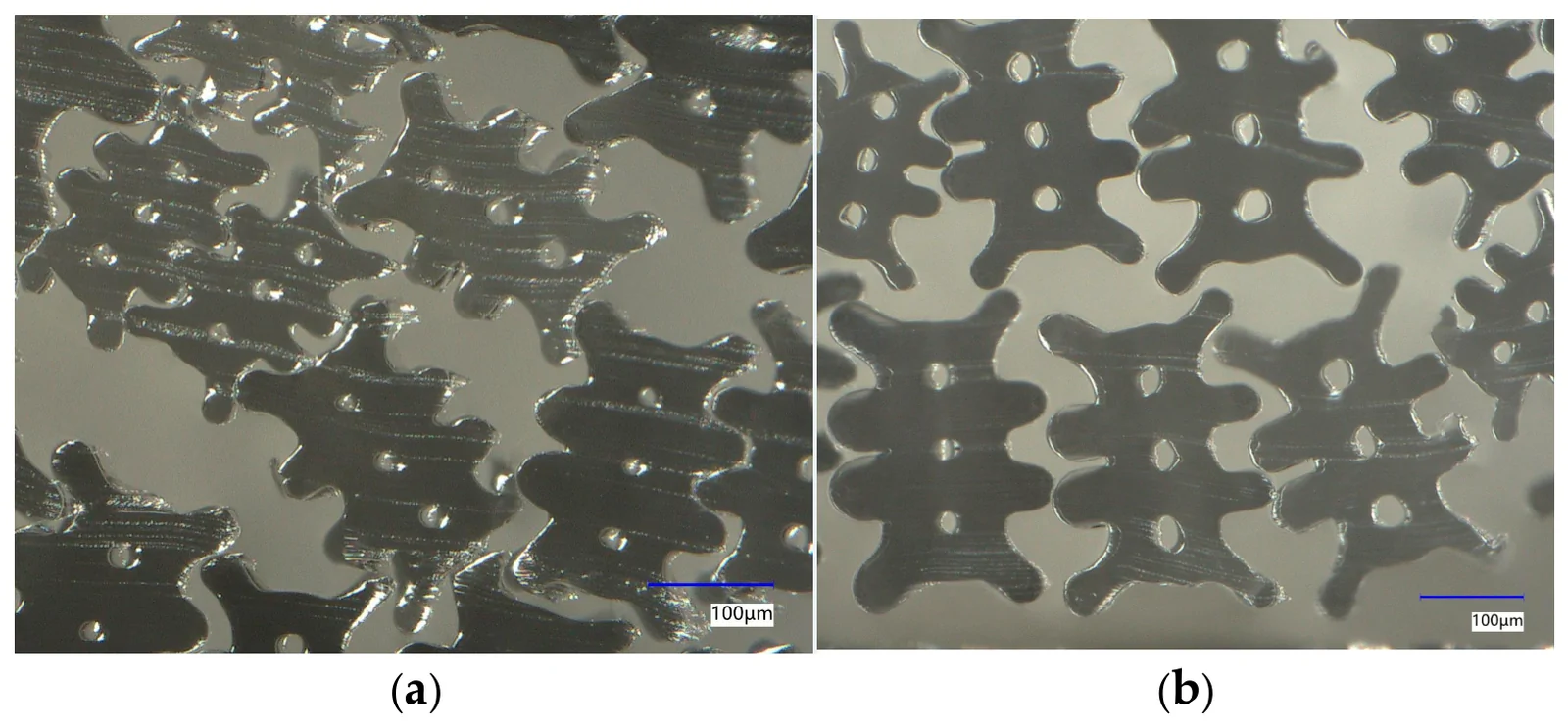
As-spun cross sections of the three-hollow eight-lobe fiber at a blowing speed of (**a**) 3.86 m/s and (**b**) 4.60 m/s. The initial quenching height was 20 mm, the quenching length was 150 mm, and the quenching air temperature was 45.2 °C. Scale bars: 100 μm.

On the basis of determining the initial blowing height of 20 mm, the influence of cooling air velocity on fiber cross-sectional forming was further studied. Six levels of blowing velocity were set, 0 m/s, 1.76 m/s, 2.35 m/s, 3.40 m/s, 3.86 m/s, and 4.60 m/s, while maintaining consistency with other process parameters. As shown in Figure 14, Figure 15 and Figure 16, in the absence of cooling air (0 m/s), the melt cooled naturally under weak convective conditions. During this prolonged low-viscosity window, surface-tension forces dominated over the flow-induced profiled deformation, causing the cross-section to tend toward a circular shape, the eight-lobe profile to be indistinct, and the hollow structure to completely fail to form. With increasing cooling air velocity, the cross-sectional shape became progressively better defined: at 1.76 m/s, the eight-lobe profile was already largely discernible and the hollow structure began to appear; at or above 2.35 m/s, the eight-lobe profile became sharper and the hollow structure tended to be fully formed.

To more precisely characterize the cross-sectional characteristics of the three-hollow eight-lobe fiber at six cooling air velocities, the degree of deviation from roundness (*D_R_*) and the hollowness ratio (*N*) were employed for quantitative evaluation. *D_R_* characterizes the extent to which the fiber cross-section deviates from a circle, whereas N is defined as the ratio of the cavity area to the total cross-sectional area. The hollowness ratio N was calculated using Equation (22):

(22) $$N=\frac{A_{1}}{A_{2}}$$

where $A_{1}$ is the cavity area (μm^2^) and $A_{2}$ is the total cross-sectional area (μm^2^). *D_R_* characterizes the extent to which the fiber cross-section deviates from a circle, while *N* is defined as the ratio of the cavity area to the total cross-sectional area.

**Figure 17:**
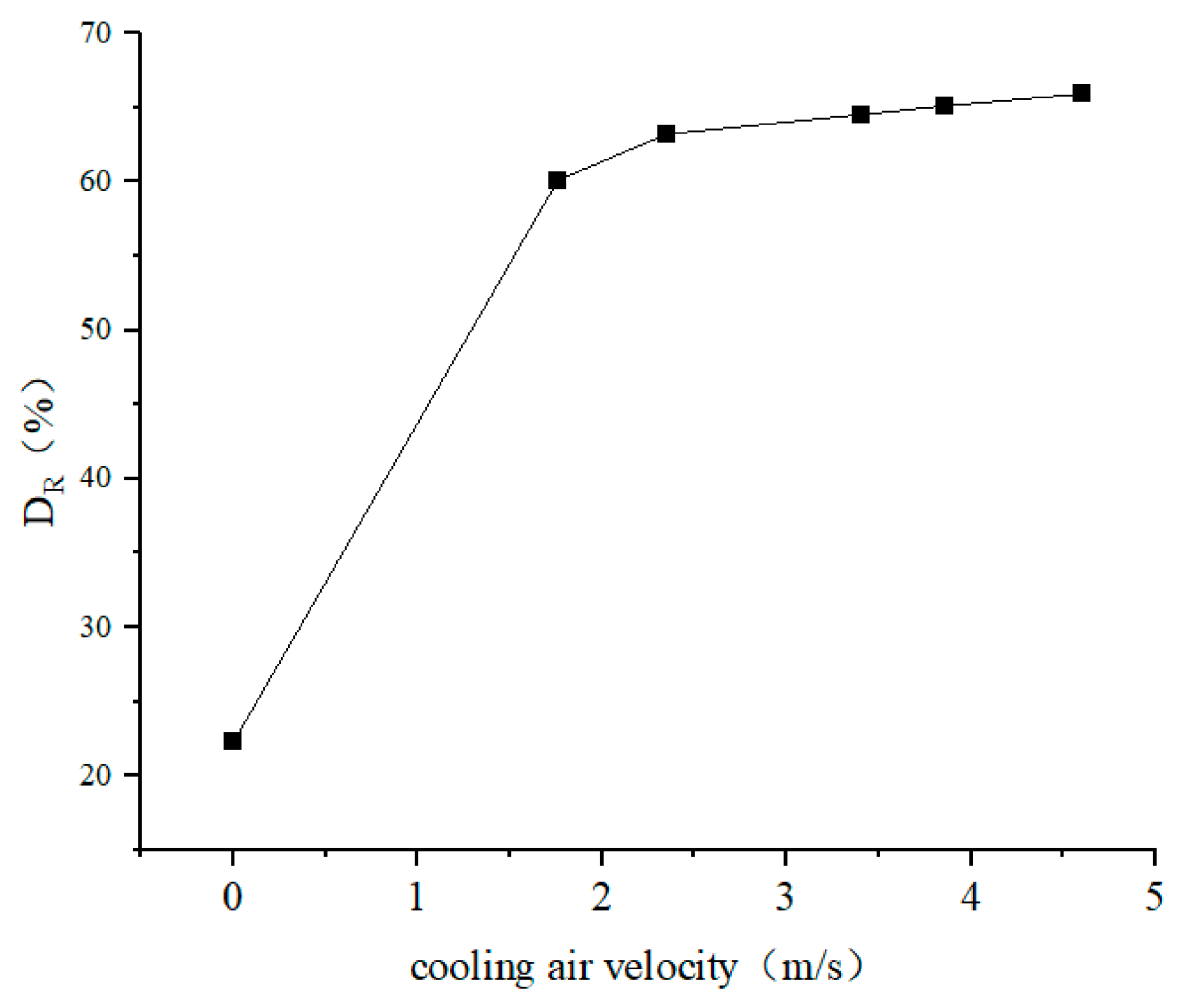
*D_R_* vs. cooling air velocity.

The calculation results show a pronounced upward trend in *D_R_* with increasing cooling air velocity. At 0 m/s, *D_R_* was only 22.3%; as the air velocity rose to 1.76 m/s, *D_R_* jumped to 60.1%. With a further increase in velocity, *D_R_* continued to rise, reaching 65.9% at 4.60 m/s. The increase in *D_R_* implies a larger specific surface area and more prominent surface grooves, which are beneficial for enhancing the capillary effect and moisture absorption and wicking properties, as shown in Figure 17.

**Figure 18:**
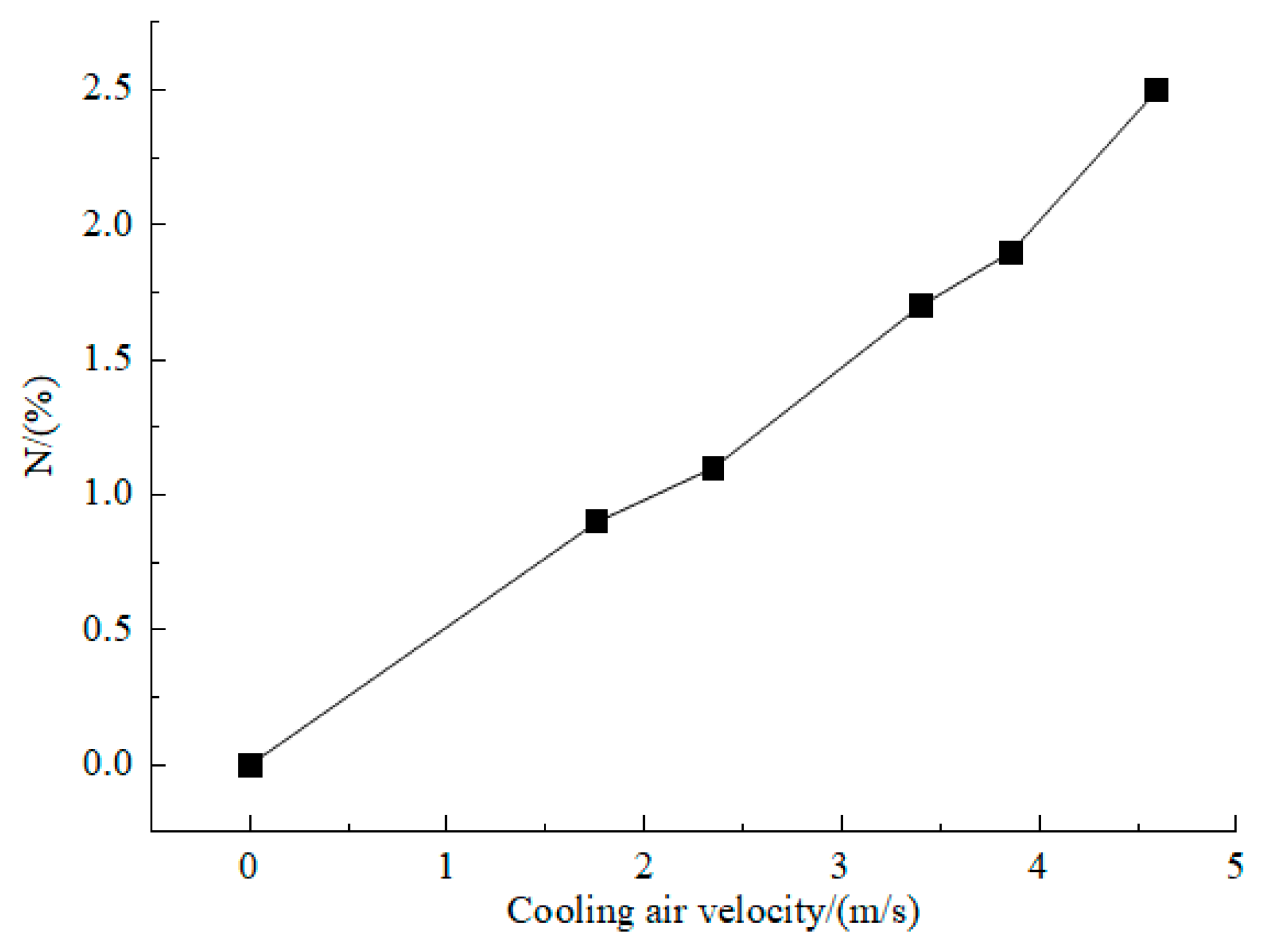
Hollowness ratio vs. cooling air velocity.

The hollowness ratio *N* also increased with increasing cooling air velocity. At 0 m/s, *N* was 0 and at 1.76 m/s, *N* was 0.9%; thereafter, *N* gradually increased to 2.5% at 4.60 m/s. The increase in hollowness helps to store more static air and enhances the thermal insulation and lightweight properties of the fibers, as shown in Figure 18.

The promoting effect of cooling air velocity on cross-section formation is governed by the competition between flow-induced deformation and surface-tension-driven shape relaxation. During spinning, the melt emerges from the profiled spinneret with a flow-induced non-circular shape and hollow channels, while surface tension simultaneously drives the cross-section toward a circular, solid form. A higher air velocity accelerates convective heat dissipation, causing the melt temperature to drop below the glass transition temperature more rapidly and triggering a sharp viscosity increase. This rapid solidification freezes the flow-induced profiled geometry before surface tension can erase it, thereby yielding higher *D_R_* and hollowness. However, at air velocities of 2.35 m/s or higher, solidification was so rapid that the rapid rise in viscosity prevented uniform accommodation of the flow-induced deformation, leading to excessive spinning stress, reduced spinnability, and eventual filament breakage. By contrast, at 0 m/s and 1.76 m/s, solidification proceeded more gradually, enabling smooth continuous winding. Therefore, 1.76 m/s was selected as the optimal compromise between cross-sectional fidelity and spinning continuity, rather than the velocity giving the maximum *D_R_*/*N*.

**Figure 19:**
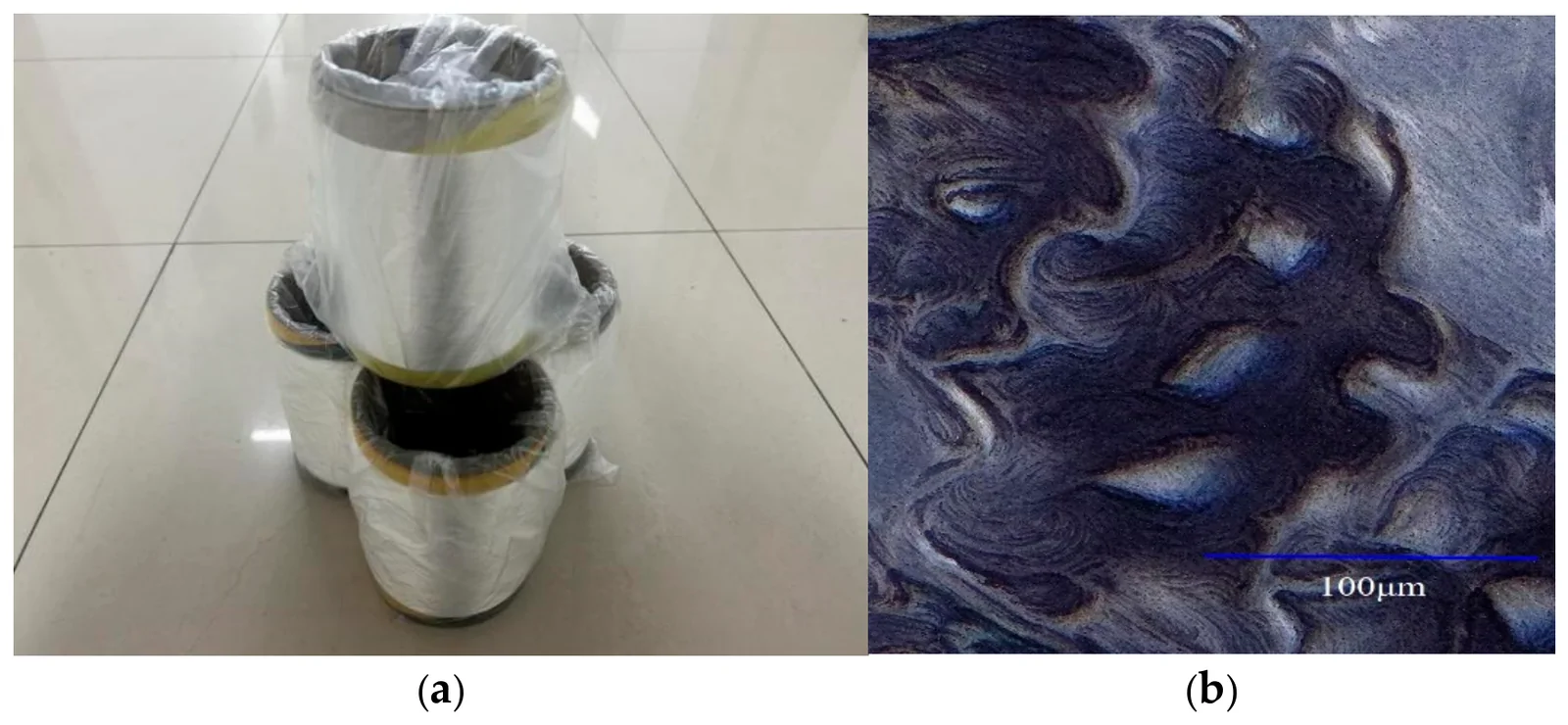
(**a**) As-wound three-hollow eight-lobe profiled fiber; (**b**) cross-section of the three-hollow eight-lobe fiber after simple drawing. The fiber was prepared at a winding speed of 2800 m/min, quenching length of 150 mm, quenching start height of 20 mm and a cooling air velocity of 1.76 m/s. Scale bars: 100 μm.

The three-hollow eight-lobe fiber after winding and simple drawing is shown in Figure 19. After simple drawing, the *D_R_* increased to 63.77%, and the linear density was 2.2 dtex, which is consistent with the design expectation.

### 4.4. Mechanical Property Testing and Statistical Analysis

Tensile fracture testing was performed on the prepared three-hollow eight-lobe fiber bundle using a yarn tensile tester. The testing conditions were as follows: initial length: 100 mm, temperature: 20 °C, relative humidity: 65%, pretension: 1 N, force attenuation rate: 80%, extension speed: 100 mm/min, and return speed: 300 mm/min. The linear density of the wound three-hollow eight-lobe fiber bundle was 127.7 dtex. Ten independent measurements were carried out (*n* = 10). Data are presented as means ± standard deviation (SD). The coefficient of variation (CV) was calculated as CV = SD/mean × 100%. The 95% confidence interval (95% CI) was calculated using the t-distribution. The test results are presented in Table 9.

The test results showed that for the three-hollow eight-lobe fiber bundle, the average breaking force was 279.2 ± 2.67 cN (95% CI: 277.3–281.1 cN; CV = 0.96%). The average breaking tenacity was 2.19 ± 0.02 cN/dtex (95% CI: 2.17–2.20 cN/dtex; CV = 0.96%). Using a PET density of 1.38 g/cm^3^, this corresponds to approximately 302 MPa (0.30 GPa). The average elongation at break was 96.4 ± 5.09% (95% CI: 92.7–100.0%; CV = 5.28%). Since the initial length was 100 mm, the elongation in mm is numerically equal to the elongation percentage. The CV of breaking force and tenacity was only 0.96%, indicating highly consistent strength. In contrast, the CV of elongation at break was 5.28%. This difference is because tenacity is mainly governed by average molecular orientation and global defect level, while elongation at break is more sensitive to local weak points, diameter fluctuation, and necking behavior. A tenacity of 2.19 cN/dtex demonstrates sufficient strength reserve to withstand tension during winding and weaving, while the elongation at break of 96.4% endows the fiber with good flexibility and deformation ability.

## 5. Fabric Preparation and Performance Testing

To evaluate the practical application performance of the three-hollow eight-lobe fiber, this study adopted a multiscale “fiber–fiber bundle–fabric” approach. The evaluation was carried out in two stages. First, simulation-based predictions were conducted at the fiber and fiber-bundle levels for six configurations. Second, based on the simulation outcomes, the three-hollow eight-lobe fiber and the eight-lobe hollow fiber were selected for experimental validation at the fabric level.

The four commercial fabrics (A–D) were used only as reference cases for fiber-bundle-scale simulations because they differed in knit structure, material composition, and construction. Therefore, they are not direct physical controls. The two experimental fibers were knitted into fabrics using the same flat knitting machine. Apart from the fiber cross-sectional shape, the two fabrics were identical in material (100% polyester), number of layers (single layer), knit structure (interlock), and stitch density. The fabric thicknesses were 0.77 mm for the three-hollow eight-lobe fabric and 0.68 mm for the eight-lobe hollow fabric.

To investigate the effect of fiber cross-sectional geometry under controlled conditions, the two fibers—identical in specification (83 dtex/36 f)—were knitted into fabrics using the same flat knitting machine, yielding the three-hollow eight-lobe fabric and the eight-lobe hollow control fabric. Apart from the difference in fiber cross-sectional shape, the two fabrics were identical in all key structural parameters: fiber material (100% polyester), number of layers (single layer), knit structure (interlock), and stitch density (wale density 11/cm, course density 12/cm). The fabric thicknesses were 0.77 mm for the three-hollow eight-lobe fabric and 0.68 mm for the eight-lobe hollow fabric, and the masses per unit area were 1.0 g/100 cm^2^ and 1.1 g/100 cm^2^, respectively. The eight-lobe hollow fiber was chosen as the experimental control because it is a widely used profiled hollow fiber in moisture-wicking textiles, thus providing a relevant comparison for investigating the effect of the novel three-hollow eight-lobe cross-section.

To evaluate the performance differences, subsequent physical testing covered three aspects: moisture wicking, anti-adhesion properties, and thermal insulation. All tests were carried out in a standard constant-temperature and constant-humidity laboratory at (20 ± 5) °C and (65 ± 2)% relative humidity to minimize environmental errors.

### 5.1. Moisture Wicking Performance Test

To dynamically assess the moisture management capacity in a simulated sweating process, a Moisture Management Tester (MMT) was employed. The instrument monitors real-time changes in electrical conductivity on both fabric surfaces, simulating dynamic moisture transport in the thickness (one-way transport) and planar (spreading) directions. The inner-surface wetting time, water absorption rate, and one-way transport index are defined as key indicators. Considering that wetting time is significantly affected by knit structure, the present experiment focused on the inner-surface water absorption rate and one-way transport index, which better reflect directional transport efficiency. A higher one-way transport index indicates stronger prevention of liquid back-flow; a higher inner-surface water absorption rate means faster uptake of transported liquid by the outer surface. The results are presented in Table 10.

The one-way transport index varied among the samples. Sportswear B exhibited the highest value (234.56), which may be related to its double-layer structure with a hydrophobic polypropylene inner layer. However, because the commercial fabrics differed in construction, they are not directly comparable to the present fabrics. Among the remaining all-polyester fabrics, the three-hollow eight-lobe fabric showed a higher one-way transport index (169.1) than the eight-lobe hollow fabric (140.1), an increase of 20.7%. The two experimental fabrics share the same single-layer interlock structure, suggesting that the difference may be related to the fiber cross-section. The inner-surface water absorption rate of the three-hollow eight-lobe fabric reached 73.67%/s, approximately 2.9 times that of the eight-lobe hollow fabric (24.65%/s). These results suggest a potential advantage in unidirectional moisture-wicking performance. However, because the number of independent fabric specimens was limited, these results are preliminary, and no statistical significance is claimed.

### 5.2. Adhesion Performance Test

The adhesion of wet fabric to skin directly affects wearing comfort. The static normal adhesion force and adhesion distance quantitatively reflect the adhesion strength and range. An XAD-1A fabric wet adhesion tester was used to simulate the peeling process and record the maximum normal adhesion force (cN) and adhesion distance (mm) [32]. A smaller force indicates weaker adhesion, and a shorter distance implies faster decay of the adhesive effect. A 0.09% saline solution was used as the test liquid, and samples were fully immersed to simulate extreme sweating conditions. Each test was repeated five times. The results are listed in Table 11.

Among the commercial controls, Sportswear B showed the lowest adhesion force (1.94 cN), which may be related to its water-repellent polypropylene inner layer. However, because their knit structures differed from those of the present fabrics, these commercial controls are not directly comparable.

Within the all-polyester group, the three-hollow eight-lobe fabric showed a lower adhesion force and a shorter adhesion distance than the eight-lobe hollow control. Its adhesion force was 3.03 cN, 24.1% lower than that of the eight-lobe hollow control (3.99 cN), and its adhesion distance was 2.2 mm, 31.3% shorter than that of the eight-lobe hollow control (3.2 mm).

The lower adhesion of the three-hollow eight-lobe fabric is unlikely to be due to fabric thickness. The two fabrics share the same material composition (100% polyester) and knit structure, which reduces differences in material and construction and helps isolate the effect of cross-sectional geometry. In addition, the three-hollow eight-lobe fabric was slightly thicker (0.77 mm vs. 0.68 mm), yet greater thickness would generally be expected to promote liquid retention and thus lead to higher rather than lower adhesion. Both its adhesion force and adhesion distance were lower. Therefore, thickness alone cannot account for the result, and the cross-sectional geometry is more likely responsible. The hollow and lobe structures contribute to anti-adhesion in different ways. The in-line three-hollow arrangement mainly increases radial compressive stiffness (Table 4), preventing fiber collapse and large-area contact with the skin. The eight-lobe structure provides surface grooves and capillary channels, maintaining discrete point and line contacts and reducing the continuity of the liquid bridge. Therefore, the combined multi-lobe, multi-hollow geometry may weaken adhesion through both mechanical support and interfacial contact control [33]. This is consistent with previous reports on the relationship between fabric geometry and adhesion force. Direct contact-area measurement and surface topography analysis were not performed in this study and are listed as future work. Nevertheless, because the number of independent fabric specimens was limited, these results are preliminary, and no statistical significance is claimed.

### 5.3. Thermal Insulation Performance Test

The thermal insulation performance was measured using a Fabric Thermal Resistance Tester. A flat specimen (300 mm × 300 mm) was placed on the heating plate (skin-contact side down) set to 35 °C, maintaining a 15 °C difference from the ambient environment. After reaching steady-state heat transfer, the instrument recorded the average heating power and calculated the clo value. Because fabric thickness, density, and surface structure significantly influence thermal resistance [34], the comparison was limited to the three-hollow eight-lobe and eight-lobe hollow fabrics, which shared identical knitting constructions. The measured clo values are listed in Table 12.

As shown in Table 12, the clo value of the three-hollow eight-lobe fabric (0.71 clo) was slightly higher than that of the eight-lobe hollow fabric (0.68 clo), corresponding to a 4.4% increase. The corresponding thermal resistance increased from 0.105 to 0.110 m^2^·K/W. Five repeated measurements were performed for each fabric, and the coefficient of variation was below 3%, indicating acceptable measurement repeatability. However, because the number of fabric specimens was limited and the two fabrics differed in thickness (0.77 mm vs. 0.68 mm), this difference cannot be attributed solely to cross-sectional geometry. The larger thickness of the three-hollow eight-lobe fabric may also contribute to its higher clo value. The three-hollow structure creates additional internal channels that may trap more static air, whose thermal conductivity is lower than that of PET. This may increase the heat conduction path and contribute to thermal resistance. Nevertheless, these results are preliminary, and no statistical significance is claimed. Further studies with controlled thickness and larger sample sizes are needed to confirm the thermal insulation advantage.

## 6. Conclusions and Discussion

In this study, a three-hollow eight-lobe PET profiled fiber was designed and fabricated through a forward-design route integrating mechanism analysis, finite element simulation, spinneret optimization, and melt spinning. The optimized cross-section consisted of eight lobes, three square hollows, and an in-line hollow arrangement. Compared with the triangular arrangement, the in-line arrangement increased the average moment of inertia by 50.8% and reduced the average deformation by 80.0%. Under the optimized spinning conditions (winding speed: 2800 m/min, quenching air speed: 1.76 m/s, initial quenching height: 20 mm, air temperature: 45.2 °C), the as-spun fiber exhibited a *D_R_* of 60.1% and a hollowness ratio of 0.9%. After simple drawing, the *D_R_* increased to 63.77% and the hollowness ratio to 8.5%. The linear density was 2.2 dtex, the tenacity was 2.19 ± 0.02 cN/dtex, and the elongation at break was 96.4 ± 5.1% (*n* = 10). In preliminary fabric-level tests, the three-hollow eight-lobe fabric showed a one-way transport index of 169.1, which was 20.7% higher than that of the eight-lobe hollow control; the adhesion force was 24.1% lower and the adhesion distance was 31.3% shorter; and the clo value was slightly higher (0.71 vs. 0.68). These results demonstrate that the three-hollow eight-lobe structure can be successfully fabricated and has potential for moisture-wicking sportswear.

However, several limitations remain. Because the number of independent fabric specimens was limited and the two fabrics differed in thickness, the fabric-level results should be considered preliminary, and no statistical significance is claimed. The initial modulus and 10% modulus could not be calculated because the raw stress–strain curves could not be obtained from the tensile tester. DMA and TGA measurements were not performed, so the thermal and dynamic mechanical stability of the fiber was not experimentally evaluated. A direct physical comparison with commercial fabrics was not conducted, and washing durability was not evaluated. In future work, full stress–strain curves will be recorded to calculate the initial modulus and 10% modulus; DMA and TGA will be performed; at least five independent specimens for each fabric will be tested; a commercial moisture-wicking fabric will be tested under identical knitting and testing conditions; and multiple washing cycles and fatigue tests will be conducted to evaluate long-term durability.

## Figures and Tables

**Table 1:** Calculation results of the degree of deviation from roundness (*D_R_*).

Fiber Type	Inscribed Circle Radius (μm)	Circumscribed Circle Radius (μm)	Degree of Deviation from Roundness (%)
Four-lobe	6.65	10.77	38.3
Five-lobe	6.43	11.19	40.0
Six-lobe	6.19	10.80	42.7
Seven-lobe	5.91	10.89	45.7
Eight-lobe	5.67	10.65	46.8

**Table 2:** Measurement results of the moment of inertia for different hollow patterns.

Hole Pattern	Moment of Inertia (μm^4^)
Single-hollow	3085.07
Two-hollow	2841.47
Three-hollow	2838.66
Four-hollow	2858.29
Five-hollow	2739.43
Six-hollow	2696.74
Seven-hollow	2676.98

**Table 3:** Maximum deformation result for different hollow patterns.

Hole Pattern	Maximum Deformation (×10^−6^ m)
Single-hollow	3.43
Two-hollow	3.05
Three-hollow	1.10
Four-hollow	0.93
Five-hollow	2.44
Six-hollow	2.52

**Table 4:** Average deformation result for different hollow arrangements.

Hollow Arrangement	Average Moment of Inertia (μm^4^)	Average Deformation (×10^−6^ m)
Triangular	2825.76	0.60
Linear	4262.36	0.12

**Table 5:** Cross-sectional shapes and linear densities of the control fibers.

Fabric	Fiber Cross-Sectional Shape	Single-Fiber Linear Density (dtex)
Sportswear A	Circular + flattened cruciform	0.69 + 0.77
Sportswear B	Circular	1.3
Sportswear C	Cruciform	1.3
Sportswear D	Circular	1.18
Eight-lobe hollow fabric	Eight-lobe hollow	1.53

**Table 6:** Number of fibers per fiber bundle.

Item (Single-Fiber Linear Density/Cross-Sectional Shape)	Number of Fibers
0.69 dtex/flattened cruciform + 0.77 dtex/circular	52 + 52
1.3 dtex/circular	60
1.3 dtex/cruciform	60
1.18 dtex/circular	65
1.53 dtex/eight-lobe hollow	50
2.2 dtex/tri-hollow eight-lobe	35

Note: For Sportswear A, the fiber bundle contains two fiber groups: 52 flattened-cruciform fibers (0.69 dtex) and 52 circular fibers (0.77 dtex), i.e., 104 fibers in total. The total bundle linear density is approximately 52 × 0.69 + 52 × 0.77 = 75.9 dtex, which is consistent with the measured bundle linear density of 76.8 dtex. For the other fiber types, the number of fibers was calculated as the bundle linear density divided by the single-fiber linear density and rounded to the nearest integer.

**Table 7:** Maximum deformation.

Number of Gaps	Gap Size (mm)	Maximum Deformation (mm)
3	0.06	0.14
3	0.08	0.12
4	0.06	0.0078
5	0.06	0.0085

**Table 8:** Mesh configurations for independence study.

Model	Cross-Sectional Partitions	Extrusion-Direction Segments	Total Elements
a	5	75	311,688
b	6	100	388,648
c	9	125	515,632

**Table 9:** Breaking test results of the three-hollow eight-lobe fiber bundle (*n* = 10).

Trial No.	Breaking Force (cN)	Tenacity (cN/dtex)	Elongation at Break (mm)	Elongation at Break (%)
1	282.2	2.2	100.2	100.2
2	273.9	2.1	87.8	87.8
3	282.2	2.2	103.4	103.4
4	279.6	2.2	97.2	97.2
5	278.3	2.2	95.7	95.7
6	280.9	2.2	99.8	99.8
7	277.4	2.1	92.5	92.5
8	281.5	2.2	101.1	
9	279.1	2.2	96.4	101.1
10	276.8	2.1	89.6	89.6
Mean ± SD	279.2 ± 2.67	2.19 ± 0.02	96.4 ± 5.09	96.4 ± 5.09
95% CI	277.3–281.1	2.17–2.20	92.7–100.0	92.7–100.0
CV (%)	0.96	0.96	5.28	5.28
Max	282.2	2.21	103.4	103.4
Min	273.9	2.14	87.8	87.8

Note: *n* = 10. Tenacity was calculated as breaking force divided by linear density (127.7 dtex). Data are means ± standard deviation (SD). 95% CI = 95% confidence interval; CV = coefficient of variation. The initial length was 100 mm, so the elongation in mm is numerically equal to the elongation percentage.

**Table 10:** Test results of the inner surface.

Fabric	Sportswear A	Sportswear B	Sportswear C	Sportswear D	Eight-Lobe Hollow	Three-Hollow Eight-Lobe
One-way transport index	81.94	234.56	149.1	140.36	140.1	169.1
Water absorption rate (%/s)	48.48	40.46	34.74	48.01	24.65	73.67

Note: The fabric-level results were obtained from a limited number of specimens and should be considered preliminary. No statistical significance is claimed. The commercial fabrics differed in knit structure and material composition from the experimental fabrics, so they are not direct controls.

**Table 11:** Measurement results of adhesion force and adhesion distance.

Fabric	Sportswear A	Sportswear B	Sportswear C	Sportswear D	Eight-Lobe Hollow	Three-Hollow Eight-Lobe
Adhesion force (cN)	5.58	1.94	6.2	4.45	3.99	3.03
Adhesion distance (mm)	28.8	17.95	26.5	22.05	3.2	2.2

Note: Data are means of five repeated measurements. The coefficients of variation for adhesion force and adhesion distance were below 5%. Because the number of independent fabric specimens was limited, these fabric-level results should be considered preliminary, and no statistical significance is claimed.

**Table 12:** Test results of fabric clo value.

Fabric	Eight-Lobe Hollow	Three-Hollow Eight-Lobe
Clo value (clo)	0.68	0.71
Thermal resistance (m^2^·K/W)	0.105	0.110

Note: The fabric-level results were obtained from a limited number of specimens and should be considered as preliminary. No statistical significance is claimed. Thermal resistance was calculated as R = clo × 0.155. The fabric thicknesses were 0.68 mm for the eight-lobe hollow fabric and 0.77 mm for the three-hollow eight-lobe fabric.

## Data Availability

The data presented in this study are available upon request from the corresponding author. For data protection reasons, the data are not publicly accessible.
